# Supplementary material for: Randomization in clinical trials with small sample sizes using group sequential designs
Source: PLoS One. 2025 Jun 13;20(6):e0325333. doi: 10.1371/journal.pone.0325333 (PMC12165385; doi:10.1371/journal.pone.0325333)
Supplement: S1 Apppendix — In this technical appendix we describe how we calculated the type I error rate and the power conditioned on a randomization sequence for boundaries from the standard Pocock, O’Brien-Fleming and Lan-DeMets designs for a z-test. (PDF) [file pone.0325333.s001.pdf]

Supplementary material to the paper:
Bodden D, Hilgers RD, König F. Randomization in clinical trials with small sample sizes using group sequential designs.

S1 Appendix. Group sequential designs using standard Pocock, O'Brien-Fleming and Lan-DeMets adjustments

We evaluated a randomized controlled trial design using a group sequential two arm parallel group design with an intended allocation ratio of 1 : 1 with continuous normally distributed endpoint. We are interested in testing the following one-sided null hypothesis on expected responses (μ_E and μ_C) $\mathrm{H}_0 : \mu_E \leq \mu_C$ against the alternative hypothesis $\mathrm{H}_1 : \mu_E > \mu_C$ using a one-sided z-test at level α with a known common variance of σ^2.

Let n_j define the number of patients allocated in stage $1 \leq j \leq K$. Consider the allocation $t_{j,i} \in \{0, 1\}$ of the i-th patient in stage j either to treatment E if $t_{j,i} = 1$ or to C if $t_{j,i} = 0$. A randomization procedure is implemented by assigning probabilities $P(T_{j,i} = t_{j,i} \mid t_{j,i} \in \{0, 1\}, 1 \leq j \leq K, 1 \leq i \leq n_j)$ to the possible allocations.

To quantify the overall response from each patient, we use a continuous, normally distributed endpoint, denoted as

$$y_{j,i} = \mu_E t_{j,i} + \mu_C(1 - t_{j,i}) + \epsilon_{j,i},$$

where $\epsilon_{j,i} \sim N(0, \sigma^2)$, $1 \leq j \leq K$, $1 \leq i \leq n_j$.

Let $n_{\mathrm{E}}(k) = \sum_{j=1}^{k} \sum_{i=1}^{n_j} t_{j,i}$ represent the number of patients allocated to treatment E up to stage k and, let $n_C(k) = \sum_{j=1}^{k} \sum_{i=1}^{n_j} (1 - t_{j,i})$ denote those allocated to treatment C. We define the mean response of patients allocated to treatments E and C up to stage k, for $1 \leq k \leq K$, as follows:

$$\bar{y}_{kE} = \frac{1}{n_{\mathrm{E}}(k)} \sum_{j=1}^{k} \sum_{i=1}^{n_j} y_{j,i} t_{j,i}, \quad \bar{y}_{kC} = \frac{1}{n_C(k)} \sum_{j=1}^{k} \sum_{i=1}^{n_j} y_{j,i} \left(1 - t_{j,i}\right)$$

for $n_{\mathrm{E}}(k) \neq 0$ and $n_C(k) \neq 0$. Therefore the first stage must include at least one allocation to each treatment group to enable the calculation of the mean for all stages. The test statistic for each stage k, where $1 \leq k \leq K$, in a two-sample z-test comparing means under a common standard deviation σ is thus given by:

$$z_k = \sqrt{I_k}\left(\bar{y}_{kE} - \bar{y}_{kC}\right)$$

where the information is defined as $\sqrt{I_k} = \left(\sigma\sqrt{\frac{1}{n_{\mathrm{E}}(k)} + \frac{1}{n_C(k)}}\right)^{-1}$.

Now, the following holds:

(i) $(z_1, z_2, \ldots, z_k)$ is multivariate normal for $1 \leq k \leq K$.

(ii) The expected value of z_k conditioned on the randomization sequence $\mathbf{T} = \mathbf{t}$ for $1 \leq k \leq K$ is given by

$$\mathrm{E}(z_k) = \sqrt{I_k}(\mu_E - \mu_C)$$

(iii) The covariance of z_k, z_l conditioned on the randomization sequence $\mathbf{T} = \mathbf{t}$ where $1 \le k \le l \le K$ is given by

$$\mathrm{Cov}(z_k, z_l) = \sqrt{I_k}\sqrt{I_l}\sigma^2.$$

The proof can be found in [1].

The information $\mathbf{I} = (I_1, \dots, I_k)$ depends on the number of allocations to group E and C in each stage and is therefore influenced by the realized allocation sequence $\mathbf{t}$.

For $\theta = \mu_E - \mu_C$, the probability of reaching the boundary at stage k, where $1 \le k \le K$, is given by

$$\Psi_k(a_1, b_1, \dots, a_k, b_k; \theta, T) = P_{\theta,T}(a_1 < z_1 < b_1, \dots, a_{k-1} < z_{k-1} < b_{k-1}, z_k > b_k).$$

Exit probabilities can be computed using the formula by Armitage et al. [2]; further calculation details are provided in [1]. Given lower (futility) boundaries $(a_1, a_2 \dots, a_{K-1})$ and upper (efficacy) boundaries $(b_1, b_2, \dots, b_K)$ the power is given by:

$$\begin{aligned} P(\text{Reject H}_0 \mid \theta, T) &= P_{\theta,Z}(z_1 > b_1) \\ &\quad + \sum_{i=2}^{K} P_{\theta,T}(a_1 < z_1 < b_1, \dots, a_{i-1} < z_{k-1} < b_{k-1}, z_K > b_K). \end{aligned}$$

Thus, using the boundaries $(a_1, b_1), \dots, (a_{K-1}, b_{K-1}), (b_K)$ from O'Brien-Fleming, Pocock, or Lan-DeMets designs, we can calculate the power of the group-sequential design for a given randomization sequence. Note that b_i denotes the efficacy boundary in stage i, while a_i (for $i \in \{1, \dots, K-1\}$) represents the futility boundary at stage i. Setting $a_i = -\infty$ implies no futility stopping is applied. When non-binding futility boundaries are used, the critical boundaries b_i are calculated with $a_i = -\infty$; however, for power and type I error calculations, the non-binding futility boundaries are used.

References

[1] Jennison, C., Turnbull, B.W.: Group Sequential Methods with Applications to Clinical Trials. Chapman & Hall/CRC Interdisciplinary Statistics. CRC Press, New York (1999)

[2] Armitage, P., McPherson, C.K., Rowe, B.C.: Repeated significance tests on accumulating data. Journal of the Royal Statistical Society. Series A (General) **132**(2), 235–244 (1969)
