# Supplementary material for: Randomization in clinical trials with small sample sizes using group sequential designs
Source: PLoS One. 2025 Jun 13;20(6):e0325333. doi: 10.1371/journal.pone.0325333 (PMC12165385; doi:10.1371/journal.pone.0325333)
Supplement: S2 Appendix — In this technical appendix we describe how we calculated the type I error rate and the power conditioned on a randomization sequence for the inverse normal combination test for a z-test. (PDF) [file pone.0325333.s002.pdf]

Supplementary material to the paper:
Bodden D, Hilgers RD, König F. Randomization in clinical trials with small sample sizes using group sequential designs.

S2 Appendix: Group sequential design with inverse normal combination test

We evaluated a randomized controlled trial design using a group sequential two arm parallel group design with an intended allocation ratio of $1:1$ with continuous normally distributed endpoint. We are interested in testing the following one-sided null hypothesis on expected responses (μ_E and μ_C) $\mathrm{H}_0 : \mu_E \leq \mu_C$ against the alternative hypothesis $\mathrm{H}_1 : \mu_E > \mu_C$ using a one-sided z-test at level α with a known common variance of σ^2.

Let n_j define the number of patients allocated in stage $1 \leq j \leq K$. Consider the allocation $t_{j,i} \in \{0,1\}$ of the i-th patient in stage j either to treatment E if $t_{j,i} = 1$ or to C if $t_{j,i} = 0$. A randomization procedure is implemented by assigning probabilities $P(T_{j,i} = t_{j,i} \mid t_{j,i} \in \{0,1\}, 1 \leq j \leq K, 1 \leq i \leq n_j)$ to the possible allocations.

To quantify the overall response from each patient, we use a continuous, normally distributed endpoint, denoted as

$$y_{j,i} = \mu_E t_{j,i} + \mu_C(1 - t_{j,i}) + \epsilon_{j,i},$$

where $\epsilon_{j,i} \sim N(0, \sigma^2)$, $1 \leq j \leq K$, $1 \leq i \leq n_j$.

Let $n_{\mathrm{E}}(k) = \sum_{j=1}^{k} \sum_{i=1}^{n_j} t_{j,i}$ represent the number of patients allocated to treatment E up to stage k and, let $n_C(k) = \sum_{j=1}^{k} \sum_{i=1}^{n_j} (1 - t_{j,i})$ denote those allocated to treatment C.

The inverse normal combination test uses the mean of each stage's test statistics instead of the cumulative mean across all previously allocated patients. This requires slight adjustments in definitions compared to the S1 Appendix.

Let $\tilde{n}_{\mathrm{E}}(k) = \sum_{i=1}^{n_k} t_{k,i}$ represent the number of patients allocated to treatment E in stage k, and let $\tilde{n}_C(k) = \sum_{i=1}^{n_j} (1 - t_{k,i})$ denote those allocated to treatment C in stage k.

The mean responses for patients in treatment group E and C in stage k (for $1 \leq k \leq K$) are defined as follows:

$$\tilde{y}_{kE} = \frac{1}{\tilde{n}_{\mathrm{E}}(k)} \sum_{i=1}^{n_k} y_{k,i} t_{k,i}, \quad \tilde{y}_{kC} = \frac{1}{\tilde{n}_C(k)} \sum_{i=1}^{n_k} y_{k,i} (1 - t_{k,i})$$

for $\tilde{n}_{\mathrm{E}}(k) \neq 0$ and $\tilde{n}_C(k) \neq 0$; thus, each stage must allocate at least one patient to both groups E and C to calculate the mean responses.

The inverse normal combination test uses a weighted test statistic that can be defined as:

$$\tilde{z}_k = \sum_{j=1}^{k} \frac{1}{\sqrt{k}} z_j^*.$$

with z_j^* given by

$$z_j^* = \sqrt{\tilde{I}_j} \left(\tilde{y}_{jE} - \tilde{y}_{jC} \right),$$

and the information defined as

$$\sqrt{\tilde{I}_j} = \left(\sigma \sqrt{\frac{1}{\tilde{n}_{\mathrm{E}}(j)} + \frac{1}{\tilde{n}_C(j)}} \right)^{-1}.$$

The expected value of $\tilde{z}_k$ is then:

$$\mathrm{E}\left(\tilde{z}_k\right) = \mathrm{E}\left(\sum_{j=1}^{k} \frac{1}{\sqrt{k}} z_j^* \right) = \frac{1}{\sqrt{k}} \left(\mu_E - \mu_C \right) \sum_{j=1}^{k} \sqrt{\tilde{I}_j}$$

for $\tilde{n}_{\mathrm{E}}(j) \neq 0$ and $\tilde{n}_{\mathrm{C}}(j) \neq 0$ for all $j \leq k$.

The covariance for the test statistics $\tilde{z}_k$ and $\tilde{z}_l$ with $k \leq l$ is:

$$\begin{aligned}
\mathrm{Cov}(\tilde{z_k}, \tilde{z_l}) &= \mathrm{Cov}\left(\sum_{j=1}^{k} \frac{1}{\sqrt{k}} z_j^*, \sum_{j=1}^{l} \frac{1}{\sqrt{l}} z_j^* \right) \\
&= \frac{1}{\sqrt{k}} \frac{1}{\sqrt{l}} \mathrm{Cov}\left(\sum_{j=1}^{k} z_j^*, \sum_{j=1}^{l} z_j^* \right) \\
&= \frac{1}{\sqrt{k}} \frac{1}{\sqrt{l}} \sum_{j=1}^{k} \tilde{I}_j \mathrm{Cov}\left(z_j^*, z_j^* \right) \\
&= \frac{1}{\sqrt{k}} \frac{1}{\sqrt{l}} \left(\sum_{j=1}^{k} \tilde{I}_j \mathrm{Cov}\left(\frac{1}{\tilde{n}_{\mathrm{E}}(j)} \sum_{i=1}^{n_j} y_{j,i} t_{j,i}, \frac{1}{\tilde{n}_{\mathrm{E}}(j)} \sum_{i=1}^{n_j} y_{j,i} t_{j,i} \right) \right. \\
&\quad \left. + \sum_{j=1}^{k} \tilde{I}_j \mathrm{Cov}\left(\frac{1}{\tilde{n}_C(j)} \sum_{i=1}^{n_j} y_{j,i} \left(1 - t_{j,i}\right), \frac{1}{\tilde{n}_C(j)} \sum_{i=1}^{n_j} y_{j,i} \left(1 - t_{j,i}\right) \right) \right) \\
&= \frac{1}{\sqrt{k}} \frac{1}{\sqrt{l}} \sum_{j=1}^{k} \tilde{I}_j \sigma^2 \left(\frac{1}{\tilde{n}_{\mathrm{E}}(j)} + \frac{1}{\tilde{n}_C(j)} \right) = \frac{1}{\sqrt{k}} \frac{1}{\sqrt{l}} k = \frac{\sqrt{k}}{\sqrt{l}}
\end{aligned}$$

for $\tilde{n}_{\mathrm{E}}(j) \neq 0$ and $\tilde{n}_{\mathrm{C}}(j) \neq 0$ for all $j \leq k$.

The covariance only exists, if all stages allocate at least one patient to both groups. When the covariance exists, it is independent of the allocation sequence. If existent, the covariance for $K = 3$ the covariance is given by:

$$\mathbf{Cov} = \begin{pmatrix} 1 & \frac{1}{\sqrt{2}} & \frac{1}{\sqrt{3}} \\ \frac{1}{\sqrt{2}} & 1 & \frac{\sqrt{2}}{\sqrt{3}} \\ \frac{1}{\sqrt{3}} & \frac{\sqrt{2}}{\sqrt{3}} & 1 \end{pmatrix}.$$

Please note, that here the link between the inverse normal combination test and group sequential design was shown assuming equal stage-wise sample sizes and a pre-planned allocation ratio of 1 : 1 (for each stage).

For $\theta = \mu_E - \mu_C$, the probability of reaching the boundary at stage $1 \leq k \leq K$ is:

$$\Psi_k\left(a_1, b_1, \ldots, a_k, b_k; \theta, T\right) = P_{\theta,T}(a_1 < \tilde{z}_1 < b_1, \ldots, a_{k-1} < \tilde{z}_{k-1} < b_{k-1}, \tilde{z}_k > b_k).$$

With this multivariate normal integral, as outlined in [1], we can calculate the power conditioned on a randomization sequence for lower (futility) boundaries $(a_1, a_2 \ldots, a_K)$ and upper (efficacy) boundaries $(b_1, b_2, \ldots, b_K)$:

$$\begin{aligned} P(\text{Reject H}_0 \mid \theta, T) = P_{\theta,T}(z_1 > b_1) \\ + \sum_{i=2}^{k} P_{\theta,T}(a_1 < \tilde{z}_1 < b_1, \ldots, a_{i-1} < \tilde{z}_{k-1} < b_{k-1}, \tilde{z}_K > b_K). \end{aligned}$$

The inverse normal combination test can apply standard group sequential boundaries. Further details are available in [2].

References

[1] Jennison, C., Turnbull, B.W.: Group Sequential Methods with Applications to Clinical Trials. Chapman & Hall/CRC Interdisciplinary Statistics. CRC Press, New York (1999)

[2] Wassmer, G., Brannath, W.: Group sequential and confirmatory adaptive designs in clinical trials. (2016)
