# Supplementary material for: Randomization in clinical trials with small sample sizes using group sequential designs
Source: PLoS One. 2025 Jun 13;20(6):e0325333. doi: 10.1371/journal.pone.0325333 (PMC12165385; doi:10.1371/journal.pone.0325333)
Supplement: S3 Appendix — This appendix provides a comprehensive overview of all simulation settings explored for the z-test, including the operating characteristics evaluated. Additionally, we detail the number of randomization sequences generated that resulted in all allocations being assigned to a single group during a stage. (PDF) [file pone.0325333.s003.pdf]

Supplementary material to the paper:
Bodden D, Hilgers RD, König F. Randomization in clinical trials with small sample sizes using group sequential designs.

S3 Appendix: Investigated values and operating characteristics of the simulation for the z-test

Table 1 Summary of design choices and assumptions used in the simulation (for the z-test).

**Name**	**Type**	**Investigated Values**
Variance	Assumption	$\sigma^2 = 1$
(Standardized) Effect size	Assumption	$\delta \in [0, 2]$ with increments of 0.2
(Planned) Allocation ratio	Assumption	1 : 1 (for each stage)
Method	Design Choice	Standard*<br>Lan-DeMets<br>Inverse Normal Combination Test
Group Sequential Boundaries	Design Choice	Pocock<br>O'Brien-Fleming
Maximum sample size n and Number of Stages K	Design Choice	$n = 12$, $K = 3$<br>$n = 24$, $K = 2$<br>$n = 24$, $K = 3$<br>$n = 48$, $K = 4$<br>$n = 120$, $K = 3$<br>$n = 12$ to $n = 60$, $K = 3$ (in increments of 6)<br>$n = 8$ to $n = 30$, $K = 3$, $K = 4$ (in increments of 1, only for permuted block randomization)
Timing of interim analysis	Design choice	Equidistant timings
Randomization procedure	Design Choice	Complete Randomization<br>Random Allocation Rule<br>Permuted Block Randomization(4)<br>Big Stick Design (3)<br>Efron's Biased Coin (2/3)<br>Chen's Design (2/3, 3)
(Overall) alpha level	Design Choice	$\alpha = 0.025$ (one-sided)
Stopping boundaries	Design Choice	One-sided with no futility<br>One-sided with non-binding futility for interim test statistic < 0<br>One-sided with binding futility for interim test statistic < 0
Alpha spending function	Design Choice	Pocock:<br>$f(t; \alpha) = \alpha \log(1 + (e - 1)t)$<br>O'Brien-Fleming:<br>$\alpha_i(t) = 2\left(1 - \Phi\left(\frac{\Phi^{-1}(\alpha/2)}{\sqrt{t}}\right)\right)$

* With standard boundaries we refer to the case where the boundaries are calculated assuming an 1 : 1 allocation ratio.

Table 2 Summary of operating characteristics evaluated in the simulation.

Name	Description
Type I error rate (T1E)	Proportion of rejections of H_0 when H_1 was false. In this paper the T1E is conditioned on the randomization sequence used.
Power	Proportion of rejections of H_0 when H_1 was true. In this paper the power of a group sequential design is conditioned on the randomization sequence. We refer to the power of a group sequential design using a specific randomization procedure as the mean power over all generated randomization sequences from the used randomization procedure.

Table 3 Allocations to a single group due to randomization. Number of allocations in the simulation of 1000 randomization sequences with a maximum sample size of $n = 24$ and $K = 3$ equidistant stages used for the power calculations. The columns represent: (2) the number of simulations in which any group received no allocations at any stage, (3) the number of simulations where one group received no allocations in the first stage and (4) the probability of assigning all patients to a single group in the first stage of the group sequential design.

Randomization procedure	Allocations to one group in any stage	Allocations to one group in stage 1	Probability of allocating all patients to a single group in the first stage
**Complete Randomization**	23	5	$2 * 0.5^8 = 1/128$
**Random Allocation Rule**	6	3	$2 * \prod_{i=0}^{7} \frac{12-i}{24-i} = 0.0013$
**Big Stick Design (m)**	0	0	0
**Permuted Block Randomization (l)**	0	0	0
**Efron's Biased Coin (p)**	4	1	$2 * 0.5 * (1/3)^7 = 0.0005$
**Chen's design (p, m)**	0	0	0
