# Supplementary material for: Randomization in clinical trials with small sample sizes using group sequential designs
Source: PLoS One. 2025 Jun 13;20(6):e0325333. doi: 10.1371/journal.pone.0325333 (PMC12165385; doi:10.1371/journal.pone.0325333)

Supplementary material to the paper:
Bodden D, Hilgers RD, König F. Randomization in clinical trials with small sample sizes using group sequential designs.

S4 Appendix: Type I error for different combinations of group sequential designs and randomization procedures using the z-test

In the "Results" section of the main manuscript, we evaluated the increase in type I error rates when standard Pocock and O'Brien-Fleming boundaries were applied for an $1:1$ allocation ratio. We presented violin plots to illustrate the type I error for each randomization sequence across various randomization procedures. Fig 1 shows the same violin plot as in the main manuscript, but starting from 0 on the y-axis.

Additionally, for validation, we assessed the type I error rate using the Lan-DeMets and inverse normal combination tests. As shown in Fig 2 and Fig 3, the type I error rate remains consistently at 0.025 across all scenarios for both designs.

Fig. 1 Violin plots showing type I error rates conditioned on the simulated randomization sequences of different randomization procedures. For a maximum sample size of $n = 24$ and three equally sized stages ($K = 3$), i.e. two interim analyses and a final analysis, using standard O'Brien-Fleming (second row) and Pocock boundaries (first row). The standard O'Brien-Fleming and Pocock boundaries assume an $1:1$ allocation at each stage. The mean type I error conditioned on the randomization sequences for each randomization procedure is shown by the red line.

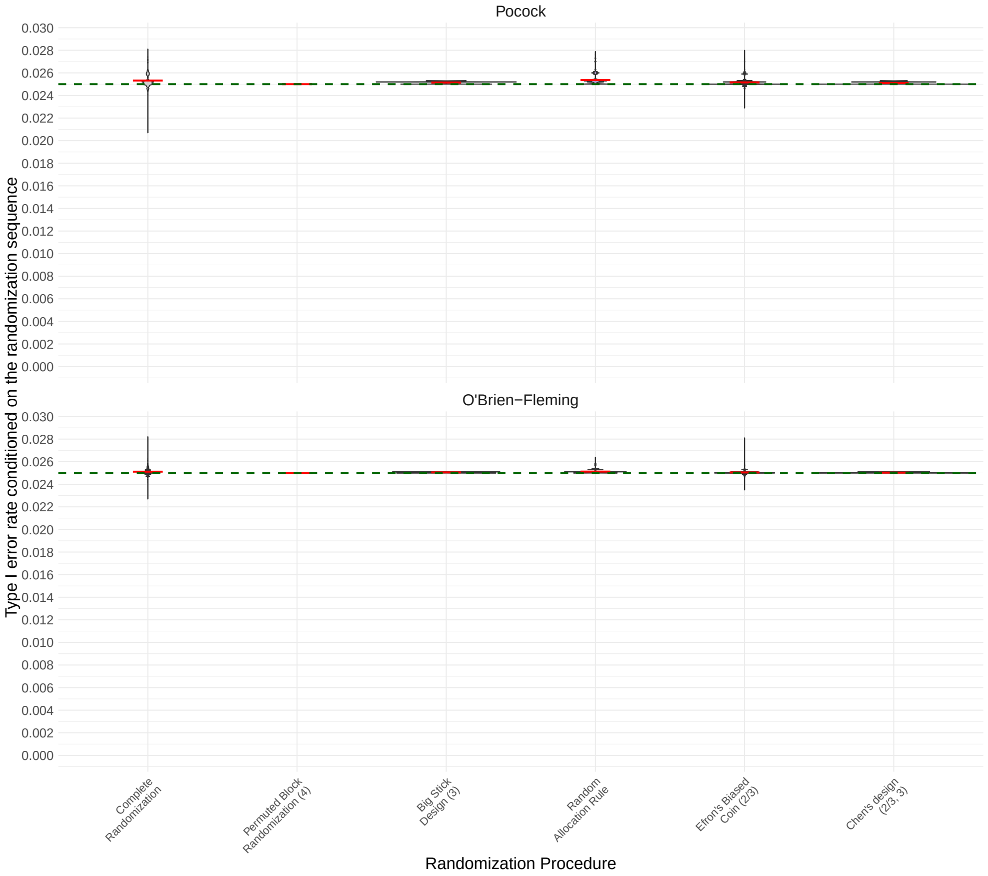

Fig. 2 Violin plots showing type I error rates conditioned on the simulated randomization sequences of different randomization procedures. For a maximum sample size of $n = 24$ and three equally sized stages ($K = 3$), i.e. two interim analyses and a final analysis, using Lan-DeMets with O'Brien-Fleming type boundaries (second row) and Pocock type boundaries (first row). The mean type I error rate conditioned on the randomization sequences for each randomization procedure is shown by the red line. Due to all data points coinciding at 0.025, the violin plots are not visible.

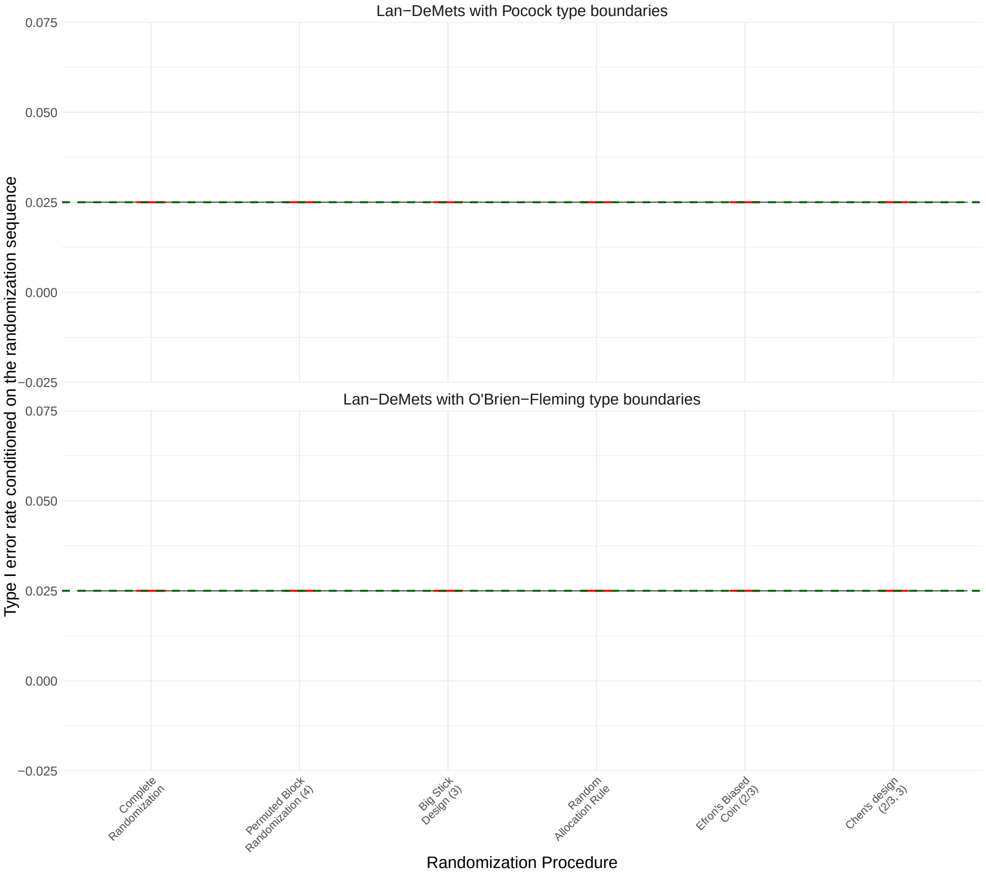

Fig. 3 Violin plots showing type I error rates conditioned on the simulated randomization sequences of different randomization procedures For a maximum sample size of $n = 24$ and three equally sized stages ($K = 3$), i.e. two interim analyses and a final analysis, using the inverse normal combination test with O'Brien-Fleming type boundaries (second row) and Pocock type boundaries (first row). Equal weights for all stages were used. The mean type I error rate conditioned on the randomization sequences for each randomization procedure is shown by the red line. Due to all data points coinciding at 0.025, the violin plots are not visible.

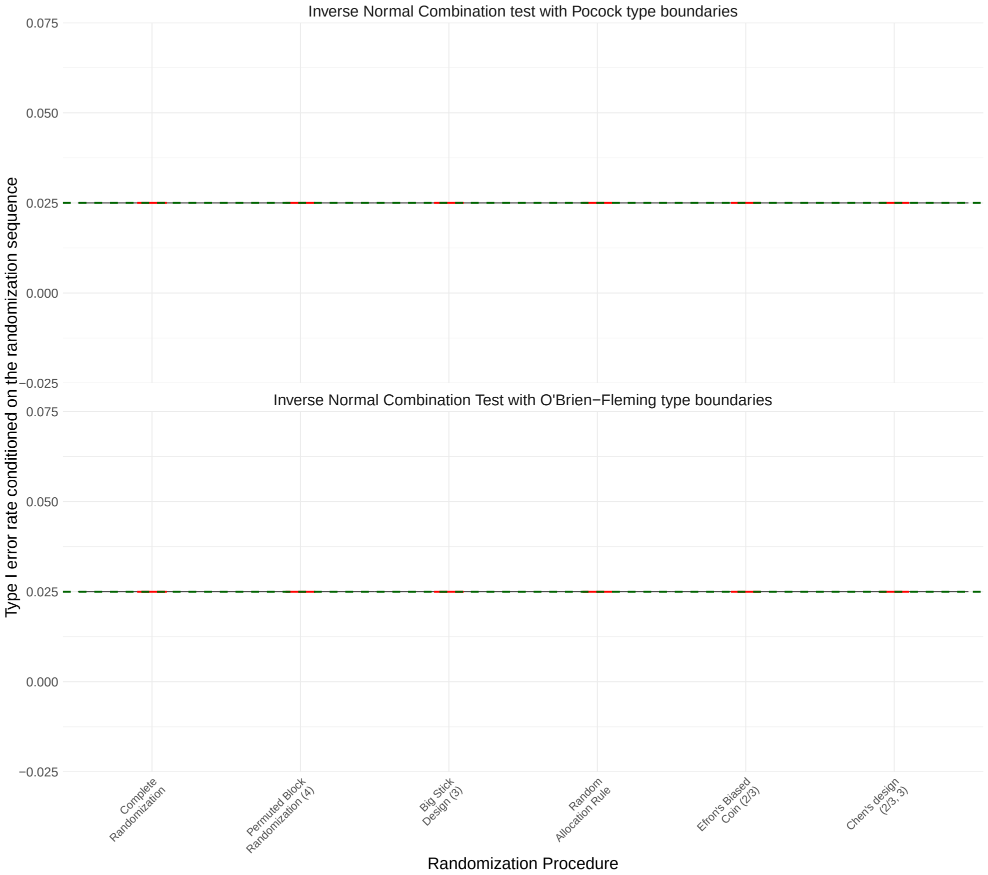

Supplement: S4 Appendix — This appendix shows the violin plots of the type I error for Lan-DeMets and inverse normal combination tests. (PDF) [file pone.0325333.s004.pdf]
