# Supplementary material for: Randomization in clinical trials with small sample sizes using group sequential designs
Source: PLoS One. 2025 Jun 13;20(6):e0325333. doi: 10.1371/journal.pone.0325333 (PMC12165385; doi:10.1371/journal.pone.0325333)

Supplementary material to the paper:
Bodden D, Hilgers RD, König F. Randomization in clinical trials with small sample sizes using group sequential designs.

S5 Appendix: Power for different combinations of group sequential designs and randomization procedures using the z-test

In the "Results" section of the main manuscript we visualized the power as a function of the effect size across different randomization procedures for the inverse normal combination test with O'Brien-Fleming type boundaries. Here we additionally show the same figures for the Lan-DeMets design, using both Pocock and O'Brien-Fleming type boundaries, as shown in Fig 1 and Fig 2, respectively. As observed, the power is generally consistent across the different randomization procedures, with the exception of complete randomization, where there is a noticeable decrease in power compared to the other procedures. In Fig 3 we show the power for the inverse normal combination test with Pocock type boundaries. The results show a substantial spread in power across the different randomization procedures, similar to the spread observed for the O'Brien-Fleming type boundaries in the inverse normal combination test presented in the main manuscript.

Fig. 1 Power as a function of effect size for a group sequential design comparing two treatment arms using Lan-DeMets with O'Brien-Fleming type boundaries. Results are shown for different randomization procedures at a nominal one-sided significance level of $\alpha = 0.025$, with a maximum sample size of $n = 24$ distributed across three equally sized stages ($K = 3$), i.e. two interim analyses and one final analysis.

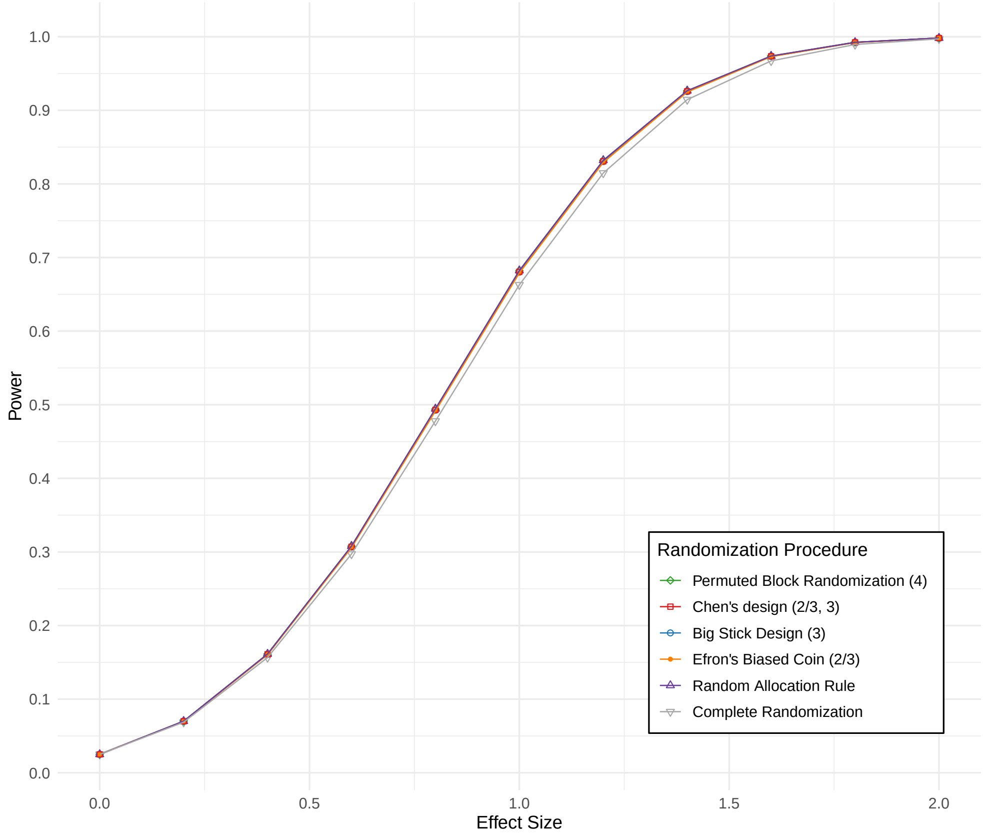

Fig. 2 Power as a function of effect size for a group sequential design comparing two treatment arms using Lan-DeMets with Pocock type boundaries. Results are shown for different randomization procedures at a nominal one-sided significance level of $\alpha = 0.025$, with a maximum sample size of $n = 24$ distributed across three equally sized stages ($K = 3$), i.e. two interim analyses and one final analysis.

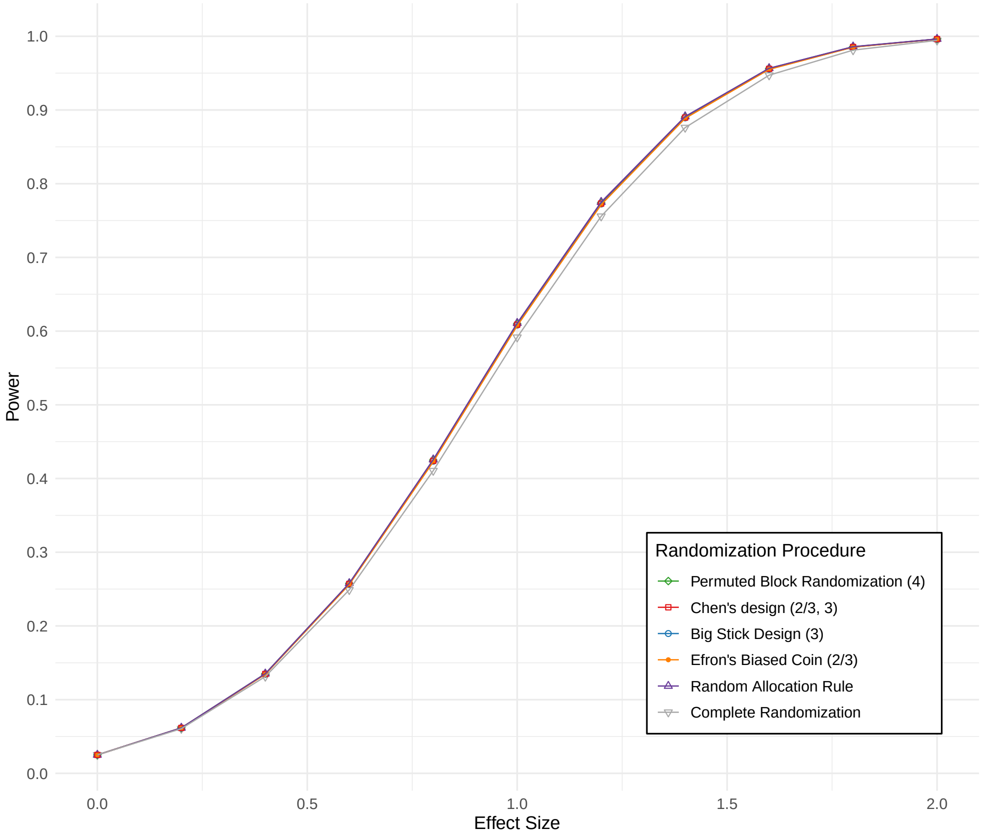

Fig. 3 Power as a function of effect size for a group sequential design comparing two treatment arms using the inverse normal combination test with Pocock type boundaries. Results are shown for different randomization procedures at a nominal one-sided significance level of $\alpha = 0.025$, with a maximum sample size of $n = 24$ distributed across three equally sized stages ($K = 3$), i.e. two interim analyses and one final analysis. Equal weights were used for the inverse normal combination test.

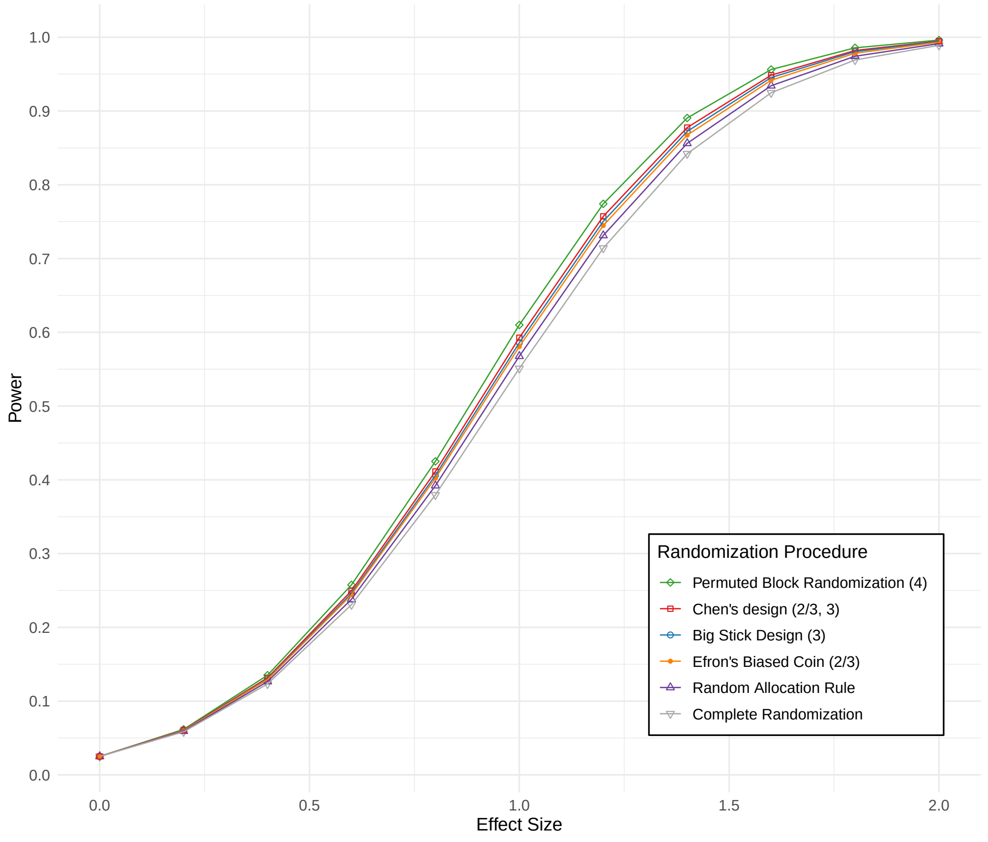

Supplement: S5 Appendix — This appendix extends the power analysis presented in the main manuscript by showing the power for additional group sequential designs. (PDF) [file pone.0325333.s005.pdf]
