# Supplementary material for: Randomization in clinical trials with small sample sizes using group sequential designs
Source: PLoS One. 2025 Jun 13;20(6):e0325333. doi: 10.1371/journal.pone.0325333 (PMC12165385; doi:10.1371/journal.pone.0325333)
Supplement: S6 Appendix — This appendix extends the power analysis presented in the main manuscript by exploring different maximum sample sizes. (PDF) [file pone.0325333.s006.pdf]

Supplementary material to the paper:
Bodden D, Hilgers RD, König F. Randomization in clinical trials with small sample sizes using group sequential designs.

# S6 Appendix: Different maximum sample sizes and number of stages for group sequential designs using the z-test

In the "Results" section of the main manuscript, we focused on a scenario involving a very small clinical trial with a maximum sample size of $n = 24$ and $K = 3$ stages. To explore the impact of different scenarios, we also evaluated various combinations of maximum sample sizes and number of stages. Our findings suggest that for smaller stage-wise sample sizes the power differences between the randomization procedures become more pronounced. This is evident in the scenario with a maximum sample size of $n = 12$ and $K = 3$ stages, as shown in Fig 1 (for Lan-DeMets with O'Brien-Fleming type boundaries) and Fig 2 (for the inverse normal combination test with O'Brien-Fleming type boundaries).

In contrast, for larger stage-wise sample sizes, these differences diminish. This can be observed in Fig 3, which presents results for a maximum sample size of $n = 48$ and $K = 4$ equally sized stages using the inverse normal combination test with O'Brien-Fleming type boundaries. Similarly, Table 1 shows the power for a maximum sample size of $n = 120$ and $K = 3$ equally distributed stages with an effect size of $\delta = 0.4$. In this scenario, we observe that only the power for complete randomization slightly decreases for Lan-DeMets. For the inverse normal combination test, both complete randomization and the random allocation rule show a slight drop in power, whereas the other evaluated randomization procedures maintain comparable power.

**Fig. 1 Power for a maximum sample size of $n = 12$ and three equally sized stages ($K = 3$) for a group sequential design comparing two treatment arms using Lan-DeMets and O'Brien-Fleming type boundaries across different randomization procedures.** For a nominal one-sided level of $\alpha = 0.025$.

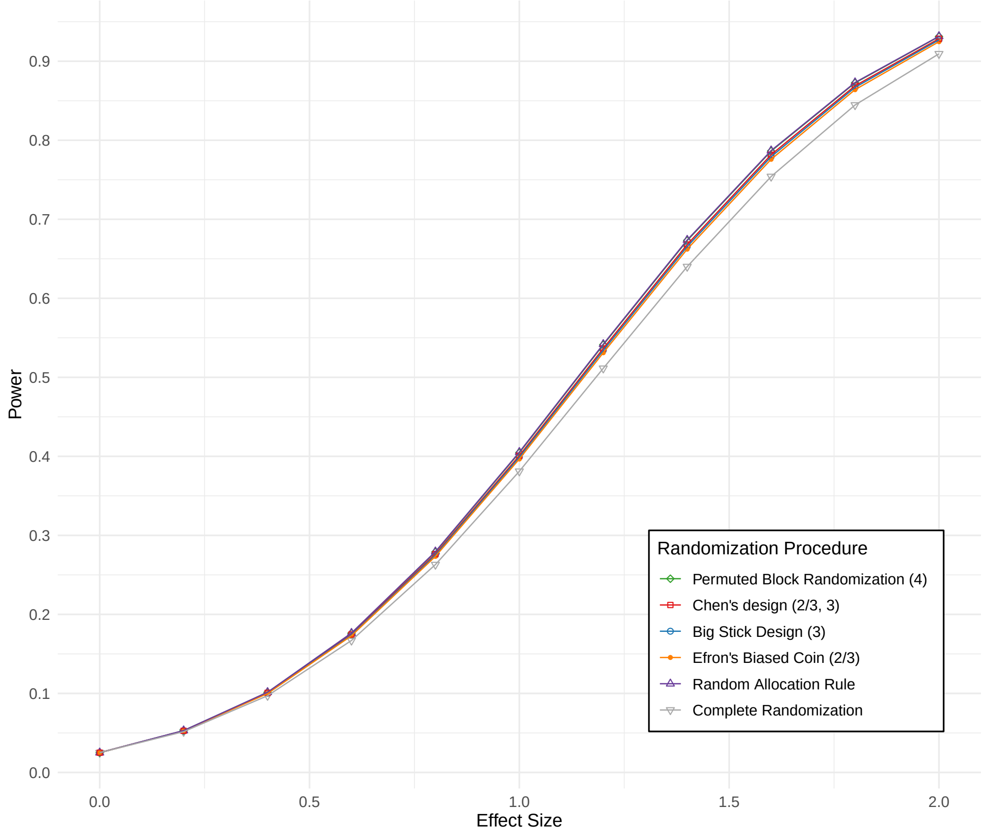

**Fig. 2 Power for a maximum sample size of $n = 12$ and three equally sized stages ($K = 3$) for a group sequential design comparing two treatment arms using inverse normal combination test and O'Brien-Fleming type boundaries across different randomization procedures.** A nominal one-sided significance level of $\alpha = 0.025$ is used, with equal weights assigned to all stages for the inverse normal combination test.

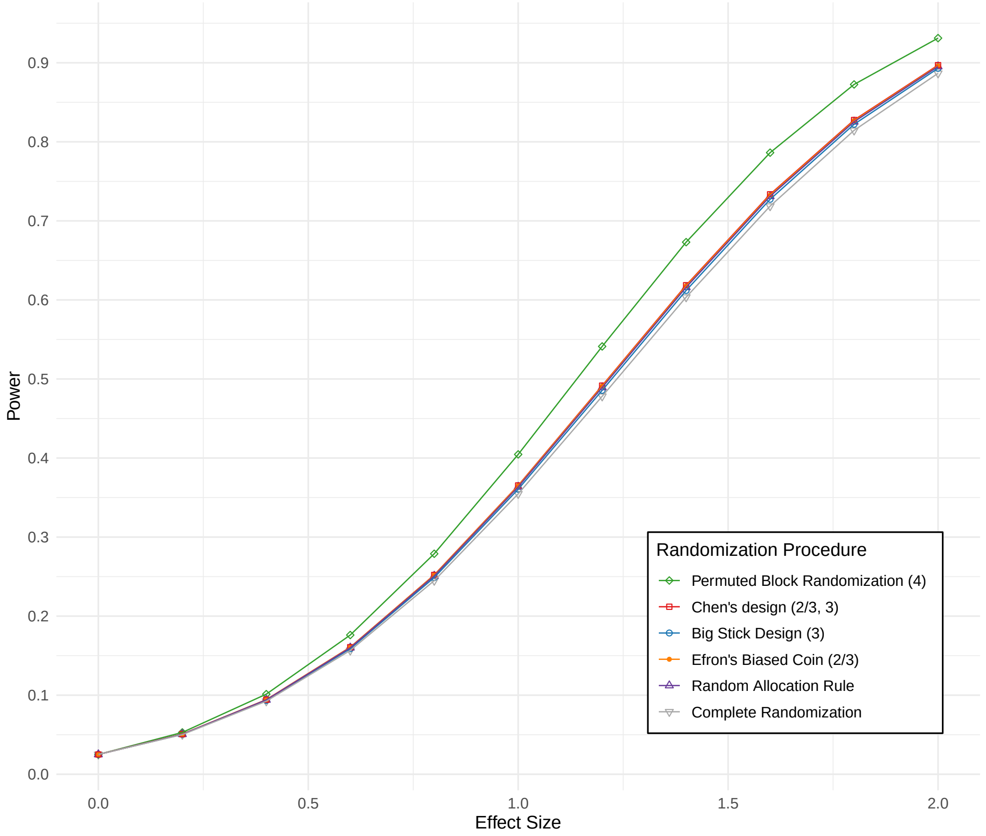

**Fig. 3 Power for a maximum sample size of $n = 48$ and four equally sized stages ($K = 4$) for a group sequential design comparing two treatment arms using inverse normal combination test and O'Brien-Fleming type boundaries across different randomization procedures.** A nominal one-sided significance level of $\alpha = 0.025$ is used, with equal weights assigned to all stages for the inverse normal combination test.

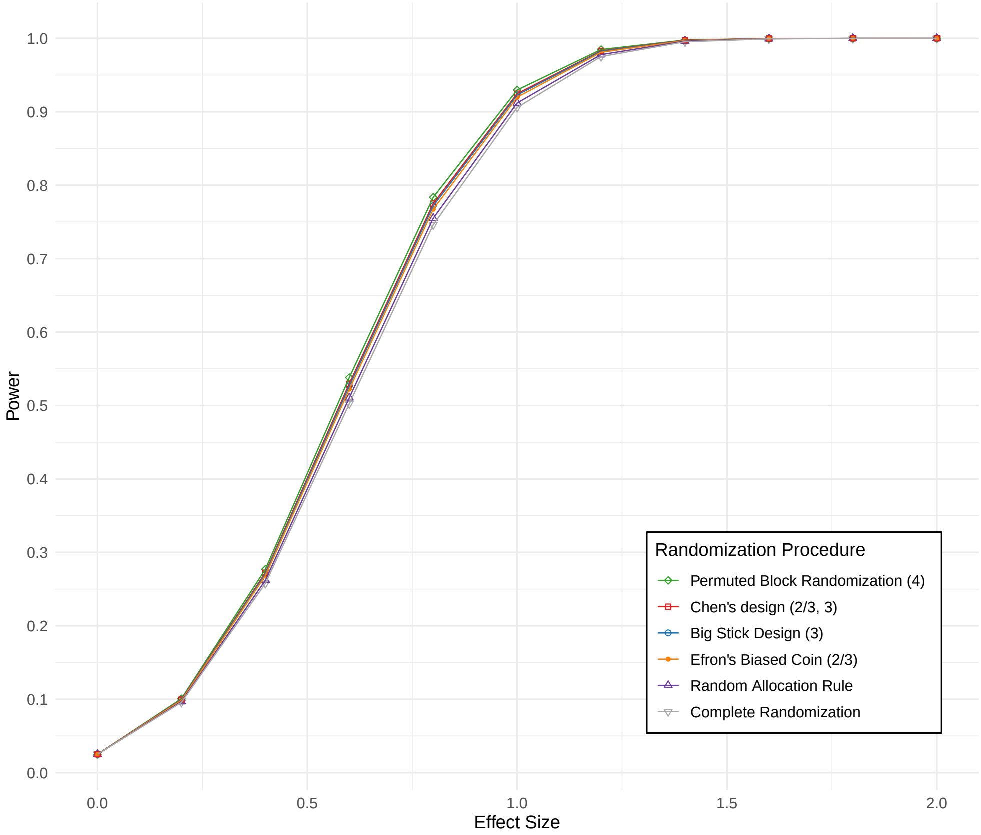

**Fig. 4 Power for a maximum sample size of $n = 120$ and three equally sized stages ($K = 3$) for a group sequential design comparing two treatment arms using inverse normal combination test and O'Brien-Fleming type boundaries across different randomization procedures.** For a nominal one-sided level of $\alpha = 0.025$. A nominal one-sided significance level of $\alpha = 0.025$ is used, with equal weights assigned to all stages for the inverse normal combination test.

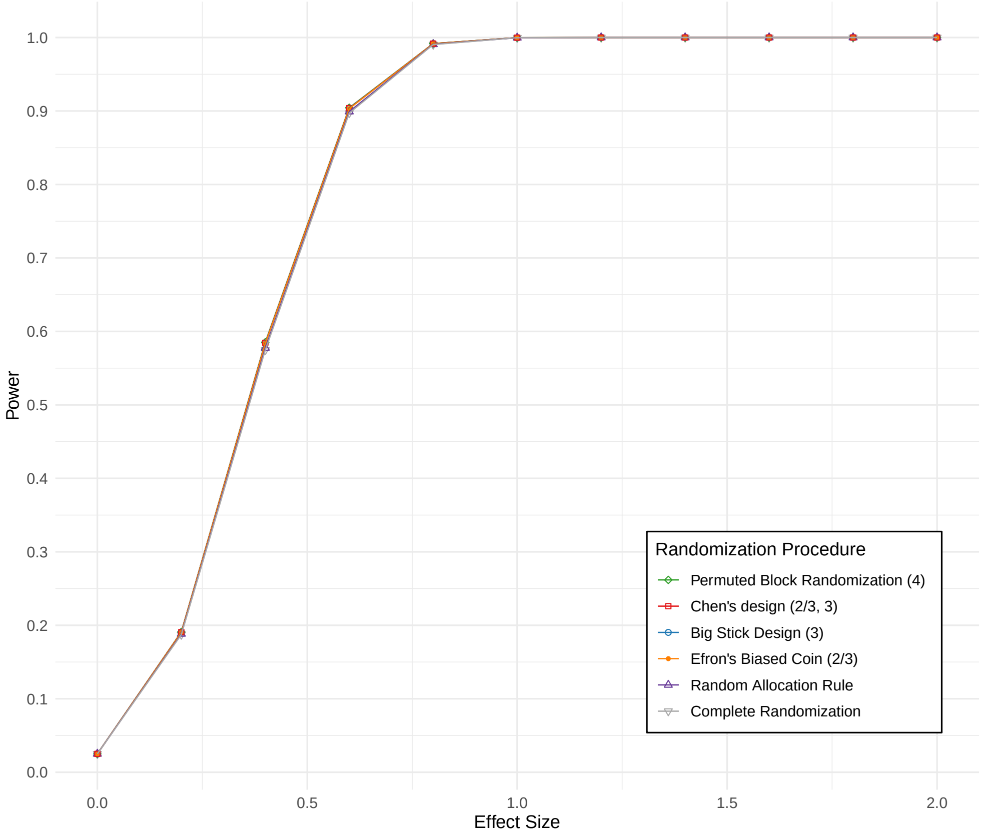

**Table 1 Power for a standardized effect size of $\delta = 0.4$ across different combinations of randomization procedures and group sequential designs.** Calculated based on 1000 randomization sequences generated from each randomization procedure to estimate the mean power. The maximum sample size is $n = 120$, distributed across three equally sized stages ($K = 3$), representing two interim analyses and one final analysis. The maximum standard error observed is 0.0003 (for the inverse normal combination test with O'Brien-Fleming type boundaries under complete randomization). For the inverse normal combination test equal weights for all stages were used.

| Randomization Procedure | Lan-DeMets with O'Brien-Fleming type boundaries | Inverse Normal Combination Test with O'Brien-Fleming type boundaries | Lan-DeMets with Pocock type boundaries | Inverse Normal Combination Test with Pocock type boundaries |
|---|---|---|---|---|
| **Complete Randomization** | 0.5819 | 0.5746 | 0.5093 | 0.5022 |
| **Permuted Block Randomization** (4) | 0.5852 | 0.5852 | 0.5125 | 0.5125 |
| **Big Stick Design** (3) | 0.5852 | 0.5841 | 0.5125 | 0.5114 |
| **Random Allocation Rule** | 0.5853 | 0.5776 | 0.5126 | 0.5051 |
| **Efron's Biased Coin** (2/3) | 0.5851 | 0.5835 | 0.5124 | 0.5109 |
| **Chen's Design** (2/3, 3) | 0.5852 | 0.5845 | 0.5125 | 0.5118 |
