# Supplementary material for: Randomization in clinical trials with small sample sizes using group sequential designs
Source: PLoS One. 2025 Jun 13;20(6):e0325333. doi: 10.1371/journal.pone.0325333 (PMC12165385; doi:10.1371/journal.pone.0325333)

Supplementary material to the paper:
Bodden D, Hilgers RD, König F. Randomization in clinical trials with small sample sizes using group sequential designs.

S7 Appendix: Permuted block randomization for different maximum sample sizes for group sequential designs using the z-test

Permuted block randomization maintains a $1 : 1$ allocation ratio for each stage only if the stage-wise sample sizes are divisible by the block size(s). This condition is achievable only for specific choices of block sizes and stage-wise sample sizes and may be disrupted by over- or under-running. We investigated the impact of not meeting this condition. To do this, we plotted the power of permuted block randomization with a block length of 4 for maximum sample sizes between $n = 8$ and $n = 30$, considering an effect size of $\delta = 1.0$ for $K = 2$, $K = 3$, and $K = 4$ stages. We evaluate both the Lan-DeMets and the inverse normal combination tests, each using O'Brien-Fleming type boundaries.

Since equidistant stages are not possible in all scenarios, we allocate $\lfloor n/K \rfloor$ patients to each stage and distribute the remaining patients consecutively, adding first to the first stage, then to the second, and so on. For example, with $n = 14$ and $K = 3$, we assign 5 patients to the first and second stages, and 4 to the third. The power for these stage-wise sample sizes is shown in Fig 1. As expected, the Lan-DeMets design remains robust to variations in stage-wise sample sizes, while the inverse normal combination test shows considerable variation in power across different maximum sample sizes. When n/K is divisible by the block size, the power of the inverse normal combination test aligns with that of the Lan-DeMets approach. However, in other scenarios, we observe a reduction in power compared to Lan-DeMets design, and in certain cases, such as $n = 13$ and $K = 3$ stages or $n = 17$ and $K = 4$ stages, we even observe lower power compared to the same design with one fewer sample. This is due to the equal weighting applied to all stages in the inverse normal combination test. We highlight the power drop for $K = 4$ between sample sizes $n = 17$ and $n = 16$ as an example of the risks of over-running in the main manuscript.

For comparison, we also evaluate a scenario where all remaining patients after allocating $\lfloor n/K \rfloor$ patients to each stage are added to the last stage, as shown in Fig 2. Here power consistently increases across all scenarios when the maximum sample size is increased, but the magnitude of the increase still varies depending on the maximum sample size.

Fig. 1 Power for $\delta = 1.0$ as a function of sample size for permuted block randomization with a block size of 4. When n/K leaves a remainder of m upon division by the block size 4, one patient is added to each of the first m stages. The sample sizes range from 8 to 30, increasing in increments of 1. For the inverse normal combination test equal weights for all stages were used.

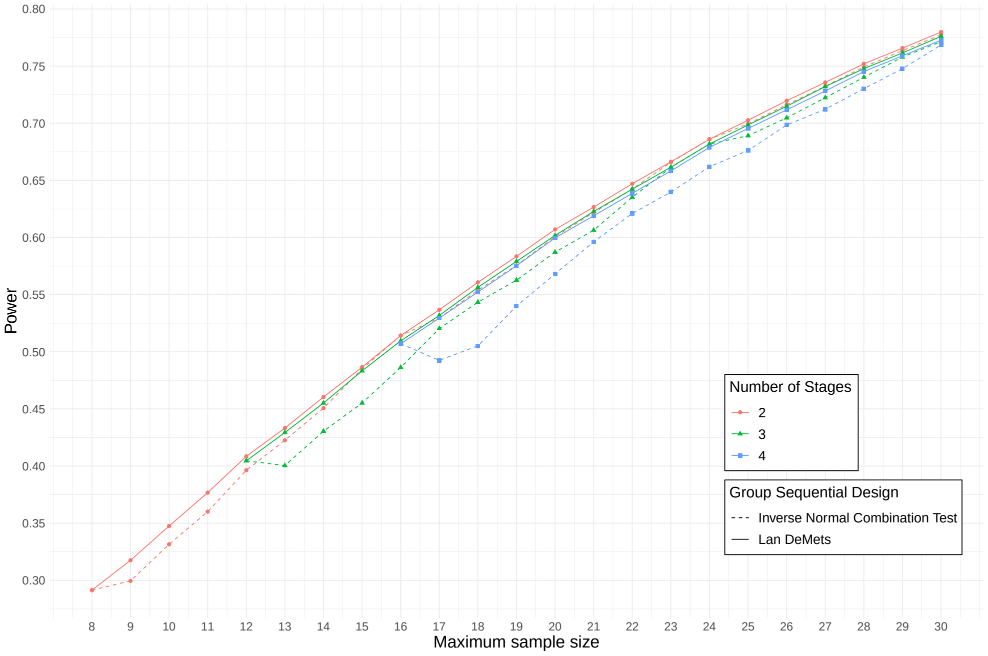

Fig. 2 Power for $\delta = 1.0$ as a function of sample size for permuted block randomization with a block size of 4. When n/K leaves a remainder of m upon division by the block size 4, the m additional patients are added to the last stage. Sample sizes range from 8 to 30, increasing in steps of 1. For the inverse normal combination test equal weights for all stages were used.

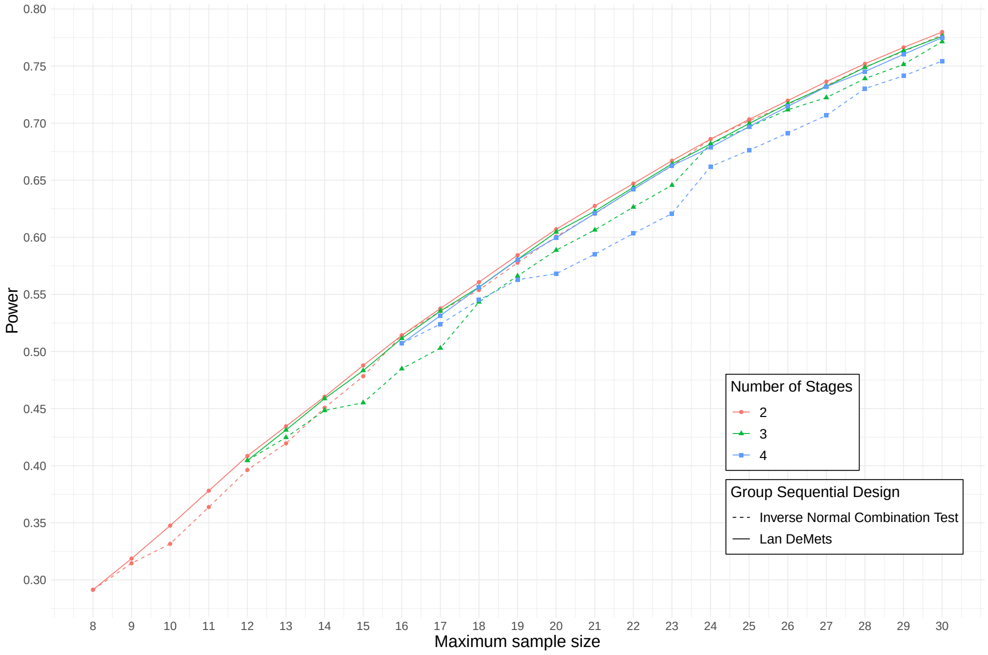

Supplement: S7 Appendix — This appendix shows the power of permuted block randomization for different maximum sample sizes, when the stage-wise sample sizes are not necessarily divisible by the block length(s). (PDF) [file pone.0325333.s007.pdf]
