# Supplementary material for: Randomization in clinical trials with small sample sizes using group sequential designs
Source: PLoS One. 2025 Jun 13;20(6):e0325333. doi: 10.1371/journal.pone.0325333 (PMC12165385; doi:10.1371/journal.pone.0325333)
Supplement: S8 Appendix — This appendix shows the methodology for the t-test in group sequential designs, describes how the simulation was conducted for the t-test, presents the type I error rate for a maximum sample size of n = 24 and K = 3 stages and presents additional results for a maximum sample size of n = 120 and K = 3 stages. (PDF) [file pone.0325333.s008.pdf]

Supplementary material to the paper:
Bodden D, Hilgers RD, König F. Randomization in clinical trials with small sample sizes using group sequential designs.

S8 Appendix: t-test for small sample group sequential designs

Following the reviewer's suggestion, we included an evaluation of the t-test in addition to the z-test in our analysis.

Study design and notation

We evaluated a randomized controlled trial design using a group sequential two arm parallel group design with an intended allocation ratio of $1:1$ with continuous normally distributed endpoint. We are interested in testing the following one-sided null hypothesis on expected responses (μ_E and μ_C) $\mathrm{H}_0 : \mu_E \leq \mu_C$ against the alternative hypothesis $\mathrm{H}_1 : \mu_E > \mu_C$ using a one-sided z-test at level α with a unknown variance σ^2.

Let n_j define the number of patients allocated in stage $1 \leq j \leq K$. Consider the allocation $t_{j,i} \in \{0, 1\}$ of the i-th patient in stage j either to treatment E if $t_{j,i} = 1$ or to C if $t_{j,i} = 0$. A randomization procedure is implemented by assigning probabilities $P(T_{j,i} = t_{j,i} \mid t_{j,i} \in \{0, 1\}, 1 \leq j \leq K, 1 \leq i \leq n_j)$ to the possible allocations.

To quantify the overall response from each patient, we use a continuous, normally distributed endpoint, denoted as

$$y_{j,i} = \mu_E t_{j,i} + \mu_C(1 - t_{j,i}) + \epsilon_{j,i},$$

where $\epsilon_{j,i} \sim N(0, \sigma^2)$, $1 \leq j \leq K$, $1 \leq i \leq n_j$.

Let $n_{\mathrm{E}}(k) = \sum_{j=1}^{k} \sum_{i=1}^{n_j} t_{j,i}$ represent the number of patients allocated to treatment E up to stage k and, let $n_C(k) = \sum_{j=1}^{k} \sum_{i=1}^{n_j} (1 - t_{j,i})$ denote those allocated to treatment C. We define the mean response of patients allocated to treatments E and C up to stage k, for $1 \leq k \leq K$, as follows:

$$\bar{y}_{kE} = \frac{1}{n_{\mathrm{E}}(k)} \sum_{j=1}^{k} \sum_{i=1}^{n_j} y_{j,i} t_{j,i}, \quad \bar{y}_{kC} = \frac{1}{n_C(k)} \sum_{j=1}^{k} \sum_{i=1}^{n_j} y_{j,i} \left(1 - t_{j,i}\right)$$

for $n_{\mathrm{E}}(k) \neq 0$ and $n_C(k) \neq 0$.

Methodology for t-test

The t-test statistic for the Lan-DeMets design is calculated as follows:

$$z_k = \frac{\bar{y}_{kE} - \bar{y}_{kC}}{\sqrt{s_p^2 \left(\frac{1}{n_E(k)} + \frac{1}{n_C(k)}\right)}}$$

where the pooled standard deviation is defined as

$$s_p(k)^2 = \frac{(n_E(k)-1)s_E^2 + (n_C(k)-1)s_C^2}{n_E(k)+n_C(k)-2}$$

with the sample variances

$$s_E(k)^2 = \frac{1}{n_E(k)-1}\sum_{i=1}^{n_j} t_{k,i}(y_{k,i}-\bar{y}_{kE}^2)$$

and

$$s_C(k)^2 = \frac{1}{n_C(k)-1}\sum_{i=1}^{n_j}(1-t_{k,i})(y_{k,i}-\bar{y}_{kC}^2)$$

for $k = 1, \dots, K$.

To compute the t-test boundaries via the quantile substitution method:

First, we transform the z-statistic boundaries $b_1, \dots, b_k$ into p-value boundaries:

$$\alpha_k = 1 - \Phi(b_k), i = 1, \dots, K$$

with Φ being the cumulative distribution function of the standard normal distribution.

Then we convert the boundaries on the p-value scale into t-statistic boundaries:

$$\tilde{b}_k = t_{\left(1-\alpha_k, \frac{kn}{K}-2\right)}$$

where $t_{\alpha,df}$ denotes the quantile function of the Student's t-distribution with df degrees of freedom.

The test decision is given by:

$$P(\text{Reject H}_0 \mid \theta, T) = P_{\theta,Z}(z_1 > \tilde{b}_1) + \sum_{i=2}^{k} P_{\theta,T}(z_1 < \tilde{b}_1, \dots, z_{k-1} < \tilde{b}_{k-1}, z_k > \tilde{b}_k).$$

Simulation Setup

Since computing the joint distribution of $(t_1, \dots, t_K)$ is hard, we use simulations instead to calculate the type I error and power for each randomization sequence. Each simulation involves:

1. Generating a randomization sequence from a given randomization procedure.
2. Simulate clinical trials to compute mean type I error and mean power for this randomization sequence.

In the same way we calculate the type I error and power for the inverse normal combination test using the stage-wise t-test statistics instead of the cumulative t-test statistics. This can be achieved by directly applying the same approach as described in S2 Appendix for the z-test.

The t-test requires that each group receives at least two allocation to perform all interim tests. Consequently, a necessary condition for conducting interim analyses using the Lan-DeMets method is that each randomization procedure assigns at least two patients to each group in the first stage. Similarly, for the inverse normal combination test, at least two patients must be allocated to each group at every stage.

If a randomization sequence failed to meet these conditions, the sequence was excluded from the analysis. Table 1 presents the number of randomization sequences where this occurred.

Table 1 Number of skipped randomization sequence for the t-test. For randomization sequences generated for a maximum sample size of $n = 24$ and $K = 3$ equidistant stages. The columns represent: (2) the number of randomization sequences generated in which any group received not more than one allocations at any stage, (3) the number of generated randomization sequences where one group received not more than one allocation in the first stage.

Randomization procedure	Randomization procedures where any group received less than two allocations in stage 1	Randomization procedures where any group received less than two allocations in any stage
**Complete Randomization**	68	206
**Random Allocation Rule**	32	75
**Big Stick Design** (m)	0	0
**Permuted Block Randomization** (l)	0	0
**Efron's Biased Coin** (p)	9	46
**Chen's design** (p, m)	0	0

Results of the simulation study for the t-test

For simulation results on power with a maximum sample size of $n = 24$ and $K = 3$ stages, we refer the reader to the section Simulation results for the t-test in the main manuscript. The corresponding type I error rates for this setting are reported in Table 4. As observed, the inverse normal combination test maintains control of the type I error rate, apart from minor deviations likely due to simulation error, whereas the Lan-DeMets approach shows inflated type I error rates, particularly when using Pocock-type boundaries. This inflation can be attributed to the quantile substitution method, which does not guarantee exact type I error control.

Additional results for $n = 120$ and $K = 3$ are presented in Table 3 for power and Table 4 for type I error rate. These findings indicate that, as the sample size increases, differences between the t-test and z-test, as well as between the various randomization procedures, become less pronounced. For the Lan-DeMets designs, power is very

Table 2 Type I error rate for each combination of randomization procedure and group sequential design for the t-test. Calculated based on 1000 randomization sequences generated from each randomization procedure and 2000 simulated trials per sequence to estimate the (mean) type I error rate. The maximum sample size is $n = 24$, distributed across three equally sized stages ($K = 3$), representing two interim analyses and one final analysis.

**Randomization Procedure**	**Lan-DeMets with O'Brien-Fleming type boundaries**	**Inverse Normal Combination Test with O'Brien-Fleming type boundaries**	**Lan-DeMets with Pocock type boundaries**	**Inverse Normal Combination Test with Pocock type boundaries**
**Complete Randomization**	0.0255	0.0251	0.0262	0.0250
**Permuted Block Randomization** (4)	0.0257	0.0251	0.0262	0.0250
**Big Stick Design** (3)	0.0257	0.0251	0.0262	0.0251
**Random Allocation Rule**	0.0258	0.0251	0.0263	0.0252
**Efron's Biased Coin** (2/3)	0.0255	0.0251	0.0260	0.0251
**Chen's Design** (2/3, 3)	0.0255	0.0250	0.0261	0.0249

similar across all randomization procedures, with only complete randomization showing slightly reduced power. The inverse normal combination test shows lower power compared to the Lan-DeMets designs, and the differences between randomization procedures are more noticeable. However, even in this case, the power differences are small and substantially attenuated compared to the $n = 24$ setting. With respect to type I error control at $n = 120$, for $K = 3$ stages, the methods appear to adequately maintain the nominal level of 2.5%.

Table 3 Power for standardized effect sizes of $\delta = 0.4$ and $\delta = 0.6$ across different combinations of randomization procedures and group sequential designs. Calculated based on 1000 randomization sequences generated from each randomization procedure and 2000 simulated trials for each randomization sequence to estimate the mean power. The maximum sample size is $n = 120$, distributed across three equally sized stages ($K = 3$), representing two interim analyses and one final analysis. For the inverse normal combination test, equal weights for all stages were used.

**Power for standardized effect size of $\delta = 0.4$**				
**Randomization Procedure**	**Lan-DeMets with O'Brien-Fleming type boundaries**	**Inverse Normal Combination Test with O'Brien-Fleming type boundaries**	**Lan-DeMets with Pocock type boundaries**	**Inverse Normal Combination Test with Pocock type boundaries**
**Complete Randomization**	0.5755	0.5635	0.4995	0.4881
**Permuted Block Randomization** (4)	0.5781	0.5743	0.5026	0.4987
**Big Stick Design** (3)	0.5784	0.5731	0.5032	0.4973
**Random Allocation Rule**	0.5784	0.5664	0.5031	0.4910
**Efron's Biased Coin** (2/3)	0.5784	0.5724	0.502	0.4968
**Chen's Design** (2/3, 3)	0.5787	0.5733	0.5031	0.4976
**Power for standardized effect size of $\delta = 0.6$**				
**Complete Randomization**	0.8975	0.8893	0.8509	0.8495
**Permuted Block Randomization** (4)	0.9000	0.8973	0.8540	0.8508
**Big Stick Design** (3)	0.8997	0.8957	0.8541	0.8486
**Random Allocation Rule**	0.8996	0.8917	0.8539	0.8434
**Efron's Biased Coin** (2/3)	0.8996	0.8957	0.8537	0.8488
**Chen's Design** (2/3, 3)	0.8995	0.8960	0.8540	0.8495

Table 4 Type I error rate for each combination of randomization procedure and group sequential design for the t-test. Calculated based on 1000 randomization sequences generated from each randomization procedure and 2000 simulated trials per sequence to estimate the (mean) type I error rate. The maximum sample size is $n = 120$, distributed across three equally sized stages ($K = 3$), representing two interim analyses and one final analysis.

**Randomization Procedure**	**Lan-DeMets with O'Brien-Fleming type boundaries**	**Inverse Normal Combination Test with O'Brien-Fleming type boundaries**	**Lan-DeMets with Pocock type boundaries**	**Inverse Normal Combination Test with Pocock type boundaries**
**Complete Randomization**	0.0250	0.0248	0.0252	0.0251
**Permuted Block Randomization** (4)	0.0252	0.0251	0.0251	0.0249
**Big Stick Design** (3)	0.0250	0.0248	0.0249	0.0248
**Random Allocation Rule**	0.0251	0.0250	0.0251	0.0250
**Efron's Biased Coin** (2/3)	0.0251	0.0248	0.0253	0.0251
**Chen's Design** (2/3, 3)	0.0251	0.0250	0.0251	0.0249
