# Supplementary material for: Randomization in clinical trials with small sample sizes using group sequential designs
Source: PLoS One. 2025 Jun 13;20(6):e0325333. doi: 10.1371/journal.pone.0325333 (PMC12165385; doi:10.1371/journal.pone.0325333)
Supplement: S9 Appendix — This appendix presents a comparison between z-test results obtained via the multivariate normal method and those derived from trial simulations. (PDF) [file pone.0325333.s009.pdf]

Supplementary material to the paper:
Bodden D, Hilgers RD, König F. Randomization in clinical trials with small sample sizes using group sequential designs.

S9 Appendix: Validation for z-test results

To validate the implementation for the z-test, we compared the results obtained via trial simulations and calculations using the multivariate normal integral. Results for power calculations are shown in Table 1, while results for type I error rates are presented in Table 2. Both methods produced similar results, supporting the validity of our implementation.

Table 1 Power for a standardized effect size of $\delta = 1.0$ across different combinations of randomization procedures and group sequential designs for the z-test for different calculation methods. Power was estimated by generating 1000 randomization sequences. For each sequence, the mean power was calculated using two methods: (i) via multivariate normal integrals (see Appendix S1 and S2) and (ii) via trial simulations, where 2000 trials were simulated for each sequence (see main manuscript, Section "Simulation study setup"). The maximum sample size was $n = 24$, distributed equally across three stages ($K = 3$), representing two interim analyses and one final analysis. For the inverse normal combination test, equal weights were used for all stages.

	**(i) Multivariate normal implementation / (ii) Trial simulation implementation**	
**Randomization Procedure**	**Lan-DeMets with O'Brien-Fleming type boundaries**	**Inverse Normal Combination Test with O'Brien-Fleming type boundaries**
**Complete Randomization**	0.6629 / 0.6629	0.6227 / 0.6227
**Permuted Block Randomization** (4)	0.6819 / 0.6823	0.6819 / 0.6811
**Big Stick Design** (3)	0.6800 / 0.6792	0.6577 / 0.6582
**Random Allocation Rule**	0.6823 / 0.6825	0.6396 / 0.6395
**Efron's Biased Coin** (2/3)	0.6790 / 0.6791	0.6517 / 0.6517
**Chen's Design** (2/3, 3)	0.6807 / 0.6806	0.6643 / 0.6646

Table 2 Type I error rate across different combinations of randomization procedures and group sequential designs for the z-test for different calculation methods. Type I error was estimated by generating 1000 randomization sequences. For each sequence, the mean power was calculated using two methods: (i) via multivariate normal integrals (see Appendix S1 and S2) and (ii) via trial simulations, where 2000 trials were simulated for each sequence (see main manuscript, Section "Simulation study setup"). The maximum sample size was $n = 24$, distributed equally across three stages ($K = 3$), representing two interim analyses and one final analysis. For the inverse normal combination test, equal weights were used for all stages.

	**(i) Multivariate normal implementation / (ii) Trial simulation implementation**	
**Randomization Procedure**	**Lan-DeMets with O'Brien-Fleming type boundaries**	**Inverse Normal Combination Test with O'Brien-Fleming type boundaries**
**Complete Randomization**	0.0250 / 0.0252	0.0250 / 0.0249
**Permuted Block Randomization** (4)	0.0250 / 0.0251	0.0250/ 0.0251
**Big Stick Design** (3)	0.0250 / 0.0251	0.0250 / 0.0251
**Random Allocation Rule**	0.0250 / 0.0251	0.0250 / 0.0251
**Efron's Biased Coin** (2/3)	0.0250 / 0.0249	0.0250 / 0.0249
**Chen's Design** (2/3, 3)	0.0250 / 0.0249	0.0250 / 0.0248
