# Supplementary material for: Randomization in clinical trials with small sample sizes using group sequential designs
Source: PLoS One. 2025 Jun 13;20(6):e0325333. doi: 10.1371/journal.pone.0325333 (PMC12165385; doi:10.1371/journal.pone.0325333)
Supplement: S10 Appendix — This appendix presents the mean absolute imbalance of group sizes for each interim and final test across different randomization procedures for a maximum sample size of n = 24 and K = 3 equidistant interim analyses. (PDF) [file pone.0325333.s010.pdf]

Supplementary material to the paper:
Bodden D, Hilgers RD, König F. Randomization in clinical trials with small sample sizes using group sequential designs.

S10 Appendix: Absolute imbalance of randomization procedure

In response to a reviewer's comment, we calculated the mean absolute imbalance for each evaluated randomization procedure for the primary analysis of a maximum sample size of $n = 24$ and $K = 3$. Table 1 presents the imbalances observed at each interim and final analysis. The results show that complete randomization leads to the highest mean imbalance at every stage. The random allocation rule has the second-highest imbalance at the first two stages but achieves perfect balance at the final stage.

The big stick design consistently shows a mean imbalance of 1.33 across all three stages, while efron's biased coin exhibits a slightly lower imbalance at each stage. Chen's design achieves the second-lowest imbalance, while permuted block randomization maintains perfect balance at every stage.

In Table 2, we further evaluated the mean absolute differences for each stage separately, as the inverse normal combination test evaluates performs the tests for each stage independently before combining the results. Here, the big stick design outperforms efron's biased coin, while the random allocation rule shows poor performance. This is because the random allocation rule only ensures overall balance, but does not control stage-wise balance.

Therefore, the results indicate that depending on the randomization procedure, restarting the randomization procedure at each stage reduces can have some benefit. However, this may result in even greater imbalance overall, which will require further investigation.

Such a stage-wise restarting approach could be considered when using inverse normal combination tests. However, this strategy is unlikely to be beneficial for designs that test the cumulative number of allocated patients rather than stage-specific patient counts. An exception is the random allocation rule, which under such conditions would effectively become equivalent to permuted block randomization with block size 8, which would also increase the predictability of the randomization procedure.

Table 1 Mean absolute difference in number of patients between treatment groups in interim and final analyses for $n = 24$ and $K = 3$ equidistant stages, by randomization procedure. The table shows the mean absolute differences in group sizes for the test performed in each stage based on 100 000 randomization sequences generated for each randomization procedure.

Randomization Procedure	Stage 1	Stage 2	Stage 3
**Complete Randomization**	2.18	3.14	3.86
**Permuted Block Randomization (4)**	0.00	0.00	0.00
**Big Stick Design (3)**	1.33	1.33	1.33
**Random Allocation Rule**	1.78	1.78	0.00
**Efron's Biased Coin (2/3)**	1.10	1.24	1.29
**Chen's Design (2/3, 3)**	0.86	0.86	0.86

Table 2 Mean absolute difference in the number of patients between treatment groups for each stage separately for $n = 24$ and $K = 3$ equidistant stages, by randomization procedure. The table shows the mean absolute differences in group sizes in each stage separatly, based on 100 000 randomization sequences generated for each randomization procedure.

Randomization Procedure	Stage 1	Stage 2	Stage 3	Sum of stage-wise differences
**Complete Randomization**	2.18	2.19	2.19	6.56
**Permuted Block Randomization (4)**	0.00	0.00	0.00	0.00
**Big Stick Design (3)**	1.33	1.36	1.36	4.05
**Random Allocation Rule**	1.78	1.77	1.78	5.33
**Efron's Biased Coin (2/3)**	1.10	1.54	1.59	4.23
**Chen's Design (2/3, 3)**	0.86	1.26	1.26	3.38
