# Supplementary material for: Randomization in clinical trials with small sample sizes using group sequential designs
Source: PLoS One. 2025 Jun 13;20(6):e0325333. doi: 10.1371/journal.pone.0325333 (PMC12165385; doi:10.1371/journal.pone.0325333)
Supplement: Attachment — Submitted filename: Response to Reviewers.pdf [file pone.0325333.s012.pdf]

POINT TO POINT ANSWERS

Manuscript ID: PONE-D-24-53742

Title: Randomization in clinical trials with small sample sizes using group sequential designs

Please find our answers to the editor and reviewers highlighted in red.

We are grateful for the thoughtful and constructive feedback, which helped us substantially improve the manuscript. We have acknowledged the editor and reviewers in the updated Acknowledgments section.

Journal requirements:

When submitting your revision, we need you to address these additional requirements.

1. Please ensure that your manuscript meets PLOS ONE's style requirements, including those for file naming. The PLOS ONE style templates can be found at

https://journals.plos.org/plosone/s/file?id=wjVg/PLOSOne_formatting_sample_main_body.pdf and

https://journals.plos.org/plosone/s/file?id=ba62/PLOSOne_formatting_sample_title_authors_affiliations.pdf

Answer: We used the PLOS ONE style template for latex. All figures and tables have been positioned immediately after the paragraph in which they are first mentioned.

2. Thank you for stating the following financial disclosure: [RDH received funding from European Rare Disease Research Coordination and Support Action consortium (ERICA) funded by the European Union's Horizon 2020 research and innovation programme under Grant Agreement. no. 964908. Further the work of FK and RDH was supported by the RealiseD project funded under Innovative Health Initiative under Grant Agreement. no. 101165912. The content of the paper reflect the personal view of the authors.]. Please state what role the funders took in the study. If the funders had no role, please state: "The funders had no role in study design, data collection and analysis, decision to publish, or preparation of the manuscript." If this statement is not correct you must amend it as needed. Please include this amended Role of Funder statement in your cover letter; we will change the online submission form on your behalf.

Answer: We have requested in the cover letter that the updated financial disclosure statement be included.

3. Thank you for uploading your study's underlying data set. Unfortunately, the repository you have noted in your Data Availability statement does not qualify as an acceptable data repository according to PLOS's standards.

At this time, please upload the minimal data set necessary to replicate your study's findings to a stable, public repository (such as figshare or Dryad) and provide us with the relevant URLs, DOIs, or accession numbers that may be used to access these data. For a list of recommended repositories and additional information on PLOS standards for data deposition, please see https://journals.plos.org/plosone/s/recommended-repositories

Answer: We have uploaded the code to a public repository on Figshare:

Bodden D, Hilgers RD, König F. Simulation code for evaluating randomization procedures in group sequential designs; 2025. DOI: 10.6084/m9.figshare.28732184.v4.

The Data Availability Statement has been updated accordingly.

Reviewer #1: Reviewer: Michael Grayling, J&J

Summary

Thanks for the opportunity to review this paper. There is a lot to like here: the authors have spotted something that has (curiously) not been discussed in the group-sequential design literature. They have then taken comprehensive approach to demonstrating this is a real issue through a literature review, and helped propose solution(s) by evaluating several possible randomisation procedures that could be utilised in practice. The manuscript is also very well written and the accompanying code is well-commented, which will heavily assist any future users. I only have one suggestion for something to add to the Discussion.

Answer: Many thanks for your encouraging feedback and appreciation of our contribution and code documentation.

Comments

1. You touch a little on assuming known variance in the Discussion, but I think you need to go slightly further on this point to help any readers understand what they'd need to do in practice in this setting. The sample sizes considered are evidently very small. In a real study, no one assumes a z-test; when using a t-test, methodology would almost certainly be needed to adjust the boundaries computed under a z-test assumption to prevent type I error inflation even in the case of perfect balance. Something like https://pubmed.ncbi.nlm.nih.gov/17434814/ or the quantile substitution approach discussed in https://pubmed.ncbi.nlm.nih.gov/18642403/ would likely be needed. So. arguably the paper is commenting on the relative inflation levels with different randomisation procedures, rather than the absolute inflation levels of each procedure (which I think could only be known with a simulation study, rather than any use of multivariate normality assumptions).

Answer: We fully agree. To address this, we added simulation results for the t-test via the quantile substitution method by trial simulations for each randomization sequence. We kept the structure of the paper showing the results for the z-test first (as in the initial submission) to highlight that there are already issues under the best assumptions, i.e., known variance. We then show how these results change when using a t-test and we provide a discussion of the differences between the two approaches.

The following sections have been added or updated:

- **Methods → New subchapter:** *Group sequential t-test*
- **Methods → Simulation study setup:** Extended to include t-test simulation details
- **Results → New subchapter:** *Simulation results for the t-test*
- **Table 6:** Power for the t-test
- **Appendix S8:** *t-test for small-sample group sequential designs* — includes the theoretical background, additional simulation results (including type 1 error rates) and a detailed methodological explanation

- **Appendix S9:** *Validation of z-test results* — compares both simulation strategies (multivariate normal vs. clinical trial simulation) for the z-test to confirm consistency of results

Within Methods sections:

Group sequential t-test

Given the small sample sizes, the t-test is generally the more appropriate choice for analysis. However, applying a t-test within a GSD is not straightforward and introduces additional challenges. To address this, we used the quantile substitution method [11]. This method transforms the z-statistic boundaries into p-values and then into t-statistic boundaries to compare them with the t-statistics calculated for the respective stages.

While this method offers a practical solution, it is important to note that it can lead to an increased T1E [35]. Details on the methodology for the group sequential t-test are provided in S8 Appendix. Alternative methods, such as determining boundaries via Monte Carlo simulation [36] or through exact calculations [9,37], are available but are rarely used due to their high computational demands.

Within simulation study setup chapter:

Following reviewers suggestion, we simulated the T1E and power when using a t-test. To achieve this, we first generated 1000 randomization sequences for each RP. Next, we simulated 2000 clinical trials and calculated the mean T1E for each randomization sequence within each RP. For LDM designs, randomization sequences, which allocated less than two patients to treatment or control group in stage 1 were skipped, as for these sequences the t-test cannot be conduced. For INCT designs, all randomization sequences that allocated less than two patients to any group and any stage were skipped, as the t-test is calculated separately for each stage for INCT. The number of excluded sequences of our simulation is provided in S8 Appendix.

Within Results section:

Results of the simulation study for the t-test

Table 6 presents the power for a standardized effect sizes of $\delta = 1.0$ and $\delta = 1.4$ across different combinations of GSDs and RPs for the t-test, analogous to Table 4 for the z-test. The results show a power loss for the t-test compared to the z-test. The overall trend in regards to the RPs is similar to the z-test, with LDM designs maintaining comparable power across most RPs except for CR, while the power difference between the RPs is more pronounced for INCT designs. For smaller sample sizes the difference between LDM and INCT is also larger, as the stage-wise calculation of t-statistics results in a higher loss of degrees of freedom compared to using LDM. The extent of the power loss between the RPs again depends on the restrictiveness of the RP.

Regarding T1E control, the t-test shows a slight inflation for a maximum sample size of $n = 24$ and $K = 3$ stages. Specifically, for the LDM design with OBF type boundaries, mean T1E up to 0.0258 is observed for the RPs and for LDM with Pocock-type boundaries, values as high as 0.0263 are reached. In contrast, the INCT controls the T1E, albeit at the cost of reduced power compared to LDM. Detailed T1E results for these settings are provided in S8 Appendix, along with additional results for a larger maximum sample size of $n = 120$. In this larger-sample scenario, the power loss associated with the t-test becomes less pronounced due to the increased degrees of freedom, and the stage-wise loss of degrees of freedom for INCT is less consequential. Likewise, the T1E inflation observed with LDM designs at $n = 24$ is mitigated with the larger sample size.

Table 6. Power for standardized effect sizes of $\delta = 1.0$ (top) and $\delta = 1.4$ (bottom) for each combination of randomization procedure and group sequential design for the t-test.

Randomization Procedure	Lan-DeMets with O'Brien-Fleming type boundaries	Inverse Normal Combination Test with O'Brien-Fleming type boundaries	Lan-DeMets with Pocock type boundaries	Inverse Normal Combination Test with Pocock type boundaries
Power for standardized effect size of $\delta = 1.0$				
Complete Randomization	0.6249	0.5719	0.5365	0.4789
Permuted Block Randomization (4)	0.6426	0.6126	0.5536	0.5178
Big Stick Design (3)	0.6398	0.5894	0.5506	0.496
Random Allocation Rule	0.6425	0.5802	0.5540	0.4864
Efron's Biased Coin (2/3)	0.6395	0.5872	0.5503	0.4944
Chen's Design (2/3, 3)	0.6410	0.5957	0.5519	0.5021
Power for standardized effect size of $\delta = 1.4$				
Complete Randomization	0.8898	0.8494	0.8311	0.7762
Permuted Block Randomization (4)	0.9021	0.8824	0.8462	0.8166
Big Stick Design (3)	0.9005	0.8644	0.8443	0.7944
Random Allocation Rule	0.9020	0.8565	0.8467	0.7844
Efron's Biased Coin (2/3)	0.9001	0.8628	0.8437	0.7926
Chen's Design (2/3, 3)	0.9008	0.8691	0.8447	0.8006

Table Description: Based on 1000 randomization sequences generated from each randomization procedure and 2000 simulated trials for each randomization sequence to calculate the (mean) power. For a maximum sample size of $n = 24$ and three equally sized stages ($K = 3$), i.e. two interim analyses and a final analysis. For a description of the randomization procedures see Table 1.

Appendix:

S8 Appendix t-test for group sequential designs. This appendix shows the methodology for the t-test in group sequential designs, describes how the simulation was conducted for the t-test, presents the type I error rate for a maximum sample size of $n = 24$ and $K = 3$ stages and presents additional results for a maximum sample size of $n = 120$ and $K = 3$ stages.

S9 Appendix Validation for z-test results. This appendix presents a comparison between z-test results obtained via the multivariate normal method and those derived from trial simulations.

----

2. Some typos I spotted:

- Abstract: “in in”
- Left quotation marks need to be inputted as `` in latex.
- Line 111: K=3 to $K=3$

Answer: Thank you for spotting these. All mentioned typos have been corrected.

Reviewer #2:

This is an interesting study. The authors investigated how randomization procedures have an impact on the results of small clinical trials using group sequential designs. Below are a few comments listed.

Small clinical trials are generally not required to have an interim analysis as the small clinical trial is not confirmatory study. It’s not very clear to me if traditional group sequential design or adaptive trial design was being studied in the paper. The traditional group sequential designs are commonly used in large clinical trials.

Answer: Thank you for this comment. We have revised the Introduction to clarify that in rare diseases, where small sample sizes and the need for efficiency are common challenges, interim analyses and group sequential designs can be particularly valuable. To support this, we have added relevant references from the rare disease research community advocating for the use of such designs, as well as regulatory guidelines that acknowledge and recommend their application in this context.

While traditional GSDs are commonly applied in larger confirmatory trials, they are increasingly being suggested for small population clinical trials, as noted by EMA’s Committee for Medicinal Products for Human Use (CHMP) guideline [1] and other published research in the field of rare diseases [2–5]. Sequential designs can be particularly advantageous in these settings by enabling early stopping for efficacy or futility, which is especially valuable when patient recruitment is challenging or when ethical considerations, such as those in orphan medicine, demand minimizing participant exposure. In some cases, treatments for rare diseases can demonstrate such pronounced efficacy that the required sample size may be considerably lower than initially anticipated [6]. In these situations, GSDs can provide an essential mechanism for early trial termination during interim analyses [4]. A systematic review of randomized clinical trials in the field of cardiovascular clinical trials [7] found that 9.3% (4 out of 43) of clinical trials with a traditional GSD had a sample size smaller than 200. ~~The~~

The authors provided the results obtained from the literature review (Table 3). The study is focused on the small clinical trials. I wonder if the sample size of each study can be added into the table to show the information.

Answer: We agree and have added the sample sizes to Table 3 and included a brief discussion of this addition in the *Results of the literature review* section.

[illegible]	[illegible]
**Planned sample size**	n = 71
1 – 100	3 (4)
101 – 200	11 (15)
201 – 500	19 (27)
501 – 1000	17 (24)
≥ 1001	20 (28)
no information	1 (1)

the same power. Most studies had a planned sample size of at least 1000, with only three trials having fewer than 101 planned participants. This highlights, despite being promoted in regulatory guidance's and methodological papers for rare-diseases, that GSDs are not widely adopted in trials with (very) small sample sizes.

P. 3, line 73. "they are particularly relevant for RD clinical trials". What does the "RD" mean?

Answer: We apologize for having used the abbreviation RD without introducing it. We have replaced the abbreviation "RD" with the full term "rare disease".

Reviewer #3:

This paper evaluate how different randomization procedures affect the type I error and power in small sample group sequential clinical trials. The topic is interesting and relevant and the paper is well written.

I have only a few minor comments:

- Abstract: In the result section: 'deficiencies in in the implementation': delete 1 'in' ; Additionally, the title of the article and the objective on p2. line 45 clearly focus on the small sample setting, which is barely mentioned in the abstract – I recommend to make it also clear in the abstract that the focus is small sample trials.

Answer: Thank you for your positive feedback. We added the focus of the small sample setting in the abstract and corrected the mentioned typo ("in in")..

Abstract

Background: Group sequential designs, which allow early stopping for efficacy or futility, may benefit from balanced sample sizes at interim and final analyses. This requirement for balance limits the choice of admissible randomization procedures. We investigate if the choice of randomization procedure, whether balanced or not, impacts the type I error probability and power in small sample clinical trials with group sequential designs.

Methods: We start with a literature review to assess how randomization procedures are reported in group sequential trials. We then investigate the impact of randomization procedures on the type I error probability and power of trials with Pocock, O'Brien-Fleming, Lan-DeMets and inverse normal combination test designs.

Results: Our findings show that only a limited number of published group sequential trials report sufficient randomization details. Simulation results demonstrate that deficiencies ~~in~~ in the implementation of randomization can inflate type I error rates. Some combinations of group sequential designs and randomization procedures cause a loss of power, for example, when using inverse normal combination tests. When the planned balanced allocation ratio in (interim) analyses cannot be ensured, the Lan-DeMets approach is preferable for small sample trials due to its robustness to deviations between the planned and observed allocation ratio. The inverse normal combination test, while useful in trials with limited prior information, should be used cautiously with permuted block randomization that maintains the planned allocation ratio to avoid power loss.

Conclusion: We propose a framework for selecting the most suitable combinations of group sequential design and randomization procedure for small sample clinical trials. Our findings highlight the need for improved reporting of randomization methods in group sequential clinical trials. Further, the validity of some of the group sequential designs relies on the application of appropriate randomization procedures which were difficult to identify in the literature and is possibly violated.

- p. 1 line 32-34: 'With respect to application of GSD's trials reported from 2001 to 2014 the LDM design was used in 34% with an OBF error spending function and 27% used the traditional OBF

design’. These percentages do not add up to 100%, which makes me wonder which error spending function is used in the remaining 39% (which is the majority of cases!).

Answer: We clarified that the reported percentages refer only to trials that disclosed their stopping boundaries. Additional types of stopping rules are mentioned in the source but not quantified. We updated the paragraph accordingly.

> Regarding the application of GSDs, a systematic review [16] of randomized controlled trials published between 2001 and 2014 found that only 65% of studies reported the stopping rules or boundaries used. Among these, 34% used the LDM design with an OBF error spending function, while 27% used the traditional OBF design. Other reported boundaries included those proposed by Pocock [11], Haybittle Peto [17], Pampallona and Tsiatis [18]. The exact percentages for the use of these designs were not specified in the systematic review [16]. ~~With respect to application of GSD’s trials reported from 2001 to 2014 the LDM design was used in 34% with an OBF error spending function and 27% used the traditional OBF design .~~

- p. 3 line 72: ‘In comparison to Stevely et al. [11], we also included randomized Phase II clinical trials utilizing GSDs, as they are particularly relevant for RD clinical trials’ – The abbreviation RD has not been introduced. Does it stand for randomized, or rare disease,…? Whatever it means, I feel the last part of that sentences is odd. If RD means randomized, then I fail to see that, if GSD is relevant for randomized clinical trials, you include only phase II trials. Additionally, do the authors mean with ‘in comparison’ that they follow the set-up of Stevely or do they mean ‘in contrast’?

Answer: We apologize for having used the abbreviation RD without introducing it. We replaced “RD” with “rare disease” and clarified that our approach contrasts with Stevely et al., not “in comparison”.

> [illegible] We combined these keywords using the Boolean operator “OR” and included the following additional eligibility criteria: human subjects, full-text, and English language. For the publication type filter, we used “Clinical trial, all”, in contrast to Stevely et. al. [16], who restricted their selection to “clinical trials, phase III”. Our broader filter was chosen to include randomized Phase II clinical trials, as these are particularly relevant for rare disease clinical trials [23]. ~~In comparison to Stevely et al. [11], we also included randomized Phase II clinical trials utilizing GSDs, as they are particularly relevant for RD clinical trials.~~ To determine the RP used in each study, we also [illegible]

- p. 3 line 76: The section that starts at this line seems out of place. Is this part of the simulation? It does not seem part of the literature review, since single center, normal endpoint trials were not part of the search criteria. Similar comment for the section starting at line 91. Perhaps they can be part of the introduction or part of the simulation section, but now these sections are disconnected and out of place.

Answer: We added a paragraph at the beginning of the Methods section outlining the structure of the subsections to help orient readers.

> **Methods**
>
> We structure the Methods section as follows: First, we describe the literature review setup. This is followed by a description of the clinical trial design and RPs. Next, we outline the adjustment for repeated interim analyses followed by how a t-test can be performed for a GSD. We conclude the section with the simulation study setup used to evaluate T1E and power. The results corresponding to each of these subsections are presented separately in the Results section.

- Table 3: GSD section, the categories sum to 67, but n=71. On p. 8 line 190-191 it is stated that ‘These percentages do not sum up to 100% as some studies utilized multiple group sequential boundaries, for instance, for different endpoints.’. If multiple methods have been used, I expect a sum

larger than 100%, not below 100%. Additionally, the ‘Information on block length’ = 14, but the below categories sum only to 13.

Answer: Thank you for your careful observation. We identified and corrected an error in the underlying Excel file related to the categorization of group sequential designs in the “Other” category. The numbers in both the table and the corresponding text have been updated accordingly.

Furthermore, only one study used multiple upper boundaries corresponding to two different primary endpoints. To clarify this, we added a footnote to the table stating that, for consistency, we report only the boundary associated with the primary endpoint deemed most relevant. The second group sequential boundary was removed from the “Other” category.

Regarding the block length information, we identified and corrected a typographical error: One study with a block length of 20 had been inadvertently omitted. This has now been corrected. We apologize for this mistake.

Table 3. Summary of results from the literature review.

Parameter	Total (%)
**Group sequential design and upper boundary**	n = 71
Lan-DeMets with O’Brien-Fleming alpha spending†	37 (52)
Lan-DeMets with Pocock alpha spending	1 (1)
O’Brien-Fleming design	9 (13)
Pocock design	2 (3)
Inverse normal combination test with O’Brien-Fleming	1 (1)
Boundaries given only	10 (14)
Other	9 (13)~~5 (7)~~
No information given	2 (3)
**Randomization Procedure**	n = 71
(Randomized) Permuted Block Randomization	30 (42)
No information on block length	16
Information on block length(s)	14
4	6
10	2
20	1
3, 6	1
4, 6	1
2, 4, 6	1
2, 4, 6, 8	1
4, 6, 8, 10	1
Minimization	9 (13)
Simple Randomization	1 (1)
No information given	31 (44)

no information 1 (1)

†One oncology study had two primary endpoints overall survival (OS) and progression free surival (PFS), each tested in the overall study population and in a pre-defined subgroup. Here we included the boundary for the more important endpoint OS, which used Lan-DeMets with O’Brien-Fleming alpha spending. For PFS a different spending function was used.

* Minimization is considered a form of stratification here.

- p. 8 line 190: based on table 3 should it not read: ‘and 3% for the Pocock design’, rather than 2%?

Answer: Thank you for noticing. Corrected.

approach with Pocock type boundaries and ~~2%~~ 3% for the Pocock design. The GSDs categorized under ”other” can be found in S11 Appendix ~~These percentages do not sum up to 100% as some studies utilized multiple group sequential boundaries, for instance, for different endpoints.~~

- p.8 line 206-207: Please revise the following sentence, which is grammatically not correct: ‘The largest inflation in mean T1E is observed for RAR, as balanced allocation at interim analysis does not always result, although RAR leads to an overall 1 : 1 allocation by the end of the trial. ‘

Answer: We have revised the sentence, thank you.

~~not maintained for every stage of the trial. The largest inflation in mean T1E is observed for RAR, as balanced allocation at interim analysis does not always result, although RAR leads to an overall 1 : 1 allocation by the end of the trial.~~
The largest inflation in mean T1E is observed for RAR, as balanced allocation at the interim analysis is not always achieved, even though RAR results in an overall 1 : 1 allocation by the end of the trial.

- p. 9 line 234: it is not clear what is meant with 2a in ‘resulting in 2a consecutive allocations to the same group’.

Answer: We have revised the sentence for clarity.

Additionally, when using RPs that allow a maximum tolerated imbalance a, this maximum tolerated imbalance should be smaller than the smallest stage size for LDM and less than half the smallest stage size for INCT. ~~This is important because an imbalance in one stage can reverse in a later stage, resulting in $2a$ consecutive allocations to the same group.~~ This requirement is necessary, because once an imbalance of a is reached in one direction, the allocation rule allows up to $2a$ consecutive allocations to the opposite group.

- p. 12 line 334: suiTable should be suitable

Answer: Corrected.

Reviewer #4:

The authors evaluated the effect of different randomization procedures on the type-I-error probability and power of a group sequential two arm parallel group design with an intended allocation ratio of 1:1 and a continuous normally distributed endpoint. The evaluation is based on an extensive simulation study combining different approaches to alpha-spending, determination of stopping boundaries, and randomization procedures for different sample sizes, using a one-tailed z-test at a one-sided level of 0.025 with a known variance of 1.

Answer: We fully agree with the summary of our paper.

Special attention is paid to small sample sizes. This raises the question of whether the z-test, which is often recommended in the literature only for sample sizes greater than 30, is appropriate. To justify this, it would have to be argued - at least by way of example - that the t-test and the z-test give comparable results even with small sample sizes, or that the evaluation is based entirely on the t-test. The R-package rpact could be helpful here.

Answer: We agree with your comment and have added dedicated t-test simulations to evaluate its impact in the small-sample setting. Please refer to our detailed response to the first answer of Reviewer #1 for a full description of the implementation and results.

Additionally, we have included a reference to the use of the rpact package.

To generate randomization sequences from various RPs, we used the R package randomizeR [40]. For group sequential boundaries, we employed the gsDesign package [41] and the rpact package [42]. The calculation of the multivariate integral needed for the simulation was performed using the mvtnorm package [43]. The

---

Another important aspect of the simulation analyses in terms of power assessment is the effect size to be detected, i.e. the number of patients required to achieve a target power or, conversely, to achieve a specific power for a given sample size. This raises the question of a justification for the choice of the effect sizes of 1.0 and 1.4 or vice versa, what effect can be detected with a sample size of 24 patients.

Answer: The effect sizes presented in the tables were selected to align with power levels close to 60% and 90% for a one-sided z-test without interim analysis under the same settings. These values were derived from a grid ranging from 0 to 2 in steps of 0.2 for the effect size. We clarified the meaning of the effect sizes in the Results section of the paper.

We next examined how these RPs influence the power of the trial under different group sequential settings. Table 4 shows the power in a setting with $n = 24$ and $K = 3$ for a standardized effect size of $\delta = 1.0$ and $\delta = 1.4$ across different combinations of RPs and GSDs. The effect size $\delta = 1.0$ corresponds to approximately 69% power for an one-sided z-test (without interim analyses) at a significance level of $\alpha = 0.025$. Similarly, an effect size of $\delta = 1.4$ corresponds to approximately 93% power under the same conditions. The power results for LDM OBF and LDM Pocock are generally similar in

---

In the discussion, the authors argued that there was a substantial power loss – due to the deviation of the intended 1:1 allocation ratio at interim analyses - but without quantifying it. It may be helpful to discuss what level of power loss is still acceptable and where it becomes so substantial that the validity of the study is questionable. Since both the power reduction and the inflated type-I-error probability are related to the deviation from the 1:1 allocation, information on the stage-wise deviation from 1:1 would be valuable.

Answer: Thank you for this valuable comment. We have removed the term “substantial” when referring to power loss, as its interpretation is context-dependent and not easily quantifiable.

Our simulation shows, that c~~C~~omplete randomization, though conceptually simple, leads to a ~~substantial~~ power loss for small sample GSDs~~, as observed in our simulations~~. To mitigate this issue, restricted randomization methods ~~like PBR~~ are commonly

To address the question of acceptable power loss, we have added the following sentence to the Discussion:

trial. ~~However, choosing an inappropriate RP can lead to substantial power loss, as our findings indicate.~~However, one should carefully evaluate if adapting the sample size is really necessary. Otherwise, LDM should be preferred in small samples due to the power loss associated with INCT compared to LDM. Furthermore, the decision which design to use and what RP to choose is accompanied by the extend of power loss acceptable, which depends on the trial settings and the cost of additional patients from a cost, availability and time perspective.

In response to your request for information on stage-wise deviations from 1:1 allocation, we simulated both the cumulative and per-stage absolute imbalances. These results have been added to Appendix S10, where (absolute) deviations from the intended 1:1 allocation ratio are now reported for each randomization procedure.

The highest power is observed for PBR(4), followed by CHEN(2/3, 3), BSD(3), EBC(2/3), RAR and finally CR. This aligns with how strictly each RP enforces balance across the stages, with more restrictive procedures maintaining higher power. The degree of restrictiveness is detailed in S10 Appendix, which presents the absolute mean differences in group sizes between the interim and final analyses for each RP.

S10 Appendix Mean absolute imbalance in number of patients for each treatment group by randomization procedure. This appendix presents the mean absolute imbalance of group sizes for each interim and final test across different randomization procedures for a maximum sample size of $n = 24$ and $K = 3$ equidistant interim analyses.

S10 Appendix: Absolute imbalance of randomization procedure

In response to a reviewer's comment, we calculated the mean absolute imbalance for each evaluated randomization procedure for the primary analysis of a maximum sample size of $n = 24$ and $K = 3$. Table 1 presents the imbalances observed at each interim and final analysis. The results show that complete randomization leads to the highest mean imbalance at every stage. The random allocation rule has the second-highest imbalance at the first two stages but achieves perfect balance at the final stage.

The big stick design consistently shows a mean imbalance of 1.33 across all three stages, while efron's biased coin exhibits a slightly lower imbalance at each stage. Chen's design achieves the second-lowest imbalance, while permuted block randomization maintains perfect balance at every stage.

In Table 2, we further evaluated the mean absolute differences for each stage separately, as the inverse normal combination test evaluates performs the tests for each stage independently before combining the results. Here, the big stick design outperforms efron's biased coin, while the random allocation rule shows poor performance. This is because the random allocation rule only ensures overall balance, but does not control stage-wise balance.

Therefore, the results indicate that depending on the randomization procedure, restarting the randomization procedure at each stage reduces can have some benefit. However, this may result in even greater imbalance overall, which will require further investigation.

Such a stage-wise restarting approach could be considered when using inverse normal combination tests. However, this strategy is unlikely to be beneficial for designs that test the cumulative number of allocated patients rather than stage-specific patient counts. An exception is the random allocation rule, which under such conditions would effectively become equivalent to permuted block randomization with block size 8, which would also increase the predictability of the randomization procedure.

Table 1 Mean absolute difference in number of patients between treatment groups in interim and final analyses for $n = 24$ and $K = 3$ equidistant stages, by randomization procedure. The table shows the mean absolute differences in group sizes for the test performed in each stage based on 100 000 randomization sequences generated for each randomization procedure.

Randomization Procedure	Stage 1	Stage 2	Stage 3
Complete Randomization	2.18	3.14	3.86
Permuted Block Randomization (4)	0.00	0.00	0.00
Big Stick Design (3)	1.33	1.33	1.33
Random Allocation Rule	1.78	1.78	0.00
Efron's Biased Coin (2/3)	1.10	1.24	1.29
Chen's Design (2/3, 3)	0.86	0.86	0.86

Table 2 Mean absolute difference in the number of patients between treatment groups for each stage separately for $n = 24$ and $K = 3$ equidistant stages, by randomization procedure. The table shows the mean absolute differences in group sizes in each stage separatly, based on 100 000 randomization sequences generated for each randomization procedure.

Randomization Procedure	Stage 1	Stage 2	Stage 3	Sum of stage-wise differences
Complete Randomization	2.18	2.19	2.19	6.56
Permuted Block Randomization (4)	0.00	0.00	0.00	0.00
Big Stick Design (3)	1.33	1.36	1.36	4.05
Random Allocation Rule	1.78	1.77	1.78	5.33
Efron's Biased Coin (2/3)	1.10	1.54	1.59	4.23
Chen's Design (2/3, 3)	0.86	1.26	1.26	3.38

The authors note that insufficiencies in the implementation of randomization can lead to an increase in the type I error rate. Furthermore, some combinations of sequential group designs and randomization procedures lead to a loss of power. To guide the selection of a suitable combinations of a sequential group design and a randomization procedure, the authors provide an overview of the combination under consideration, together with a traffic light rating. This framework seems to be the key message of the manuscript and should be pronounced more strongly. Further explanations and practical recommendations (in addition to the bullet list on page 12) would be useful. E.g. information on type-I-error probability and power for each combination (even if it can be seen in the appendix, it would be nice for a "framework" to have all the information bundled in one place). Another point would be, for example that for the Random Allocation Rule and Efron's Biased Coin Design the "risk of allocations to single group" is only present if the randomization procedure is applied over all stages. This risk cannot occur if the procedure is implemented stages-wise.

Answer: Thank you for this helpful suggestion. We have moved the traffic-light framework and the bullet-point recommendations to a new dedicated subsection at the end of the Results section. We also expanded the bullet-point recommendations to include key findings from both the main manuscript and the supplementary analyses presented in the appendix.

Framework for selecting the randomization procedure in small sample group sequential designs

Based on our simulation studies, we summarize key considerations we found during our simulations:

1. When using test statistics based on cumulative data with a pre-defined spending function one has to account for the observed allocation ratio and the potential stage-wise imbalances when calculating the actual critical boundaries toensure the T1E is controlled even for the z-test (assuming known variances).
2. Avoid RPs that can result in arbitrarily large imbalances within stages, such as CR, EBC and RAR.
3. If the correct spending function incorporating the observed allocation ratio is used, then the power of LDM designs remains robust when using RPs that do not maintain an exact 1 : 1 stage-wise allocation ratio, but achieve a sufficiently close balance, e.g., there is a loss of power for complete randomization (especially for moderate effect sizes).
4. For LDM T1E control becomes tricky. Even when using the quantile substitution method for the t-test the T1E may be inflated, particularly for very small sample sizes, see S8 Appendix.
5. Using the INCT strictly controls the T1E both for z-test and t-test. However, there is a trade-off between strict T1E control and power.
6. When using INCT, RPs that closely maintain the stage-wise 1 : 1 allocation ratio should be preferred to maintain power. For example, when using PBR, select a

block size that is a divisor of the stage-wise sample size to meet the targeted allocation ratio and maintain power.

7. For the t-test the INCT has a loss in power compared to LDM. This is more pronounced for smaller sample sizes as in the t-test more degrees of freedom have to be spent for INCT to calculate stage-wise variances and test statistics.

8. For larger stage-wise sample sizes, differences in power, both in regards to the RP and t-test vs. z-test become less relevant (see S6 Appendix, S8 Appendix). However, the INCT would allow for additional features, e.g., sample size reassessment if needed.

~~A summary of the findings includes the following points:~~
~~-RPs that can lead to significant imbalance, such as RAR, CR or EBC, should be avoided for small stage-wise sample sizes due to the lower power and possibility of allocations to only a single treatment group in a stage.~~
~~-The power of LDM designs is robust in regards to different RPs that do not necessarily maintain the intended 1 : 1 allocation ratio (for each stage).~~
~~-When INCT designs are used, strict adherence to stage-wise sample sizes is necessary to avoid a power drop due to the weights being chosen for an intended 1 : 1 allocation ratio.~~
~~-PBR should only be used with a block size that is a divisor of the stage-wise sample sizes to maintain high power.~~

In light of these findings, it becomes clear that the choice of RP in combination with the GSD is important in small sample settings. To guide these decisions, we propose a framework that synthesizes the insights from our analysis, including considerations on allocation imbalances, power differences across RPs, and the conditions under which PBR must be aligned with stage-wise sample sizes for optimal power. Table 7 shows this framework, which is relevant for small stage-wise sample sizes, say a (minimum) stage-wise sample size that is smaller than 20.

To address the point about stage-wise implementation, we clarified in the Methods section that the randomization procedures evaluated in this study are applied across the entire trial, rather than re-initialized at each stage.

in stratified randomization [24]. Table 1 provides an overview of key terms related to randomization in GSDs. Specifically, the RPs evaluated in this study are defined, providing a reference for the methods under consideration. The RPs will be applied across the entire trial rather than being implemented separately for each stage.

Additionally, based on the simulation results presented in Appendix S10, we added a recommendation that, for inverse normal combination tests, restarting the randomization procedure at each stage may help mitigate imbalances and improve performance.

Some RPs can be modified, so that their restrictions apply for each stage to enforce a close 1 : 1 balance not overall, but stage-wise, as in stratified randomization. However the modifications have to be implemented in such a way that the overall balance is not negatively affected. For example if the same maximum tolerated imbalance would be applied in a stage-wise manner, the actual tolerated one would accumulate. The disadvantage is, that this leads to even more restrictive RPs. Enforcing a 1 : 1 allocation at the end of each stage is mathematically equivalent to employing PBR with a block size equal to the stage size as an additional restriction, which increases the predictability and allocation bias risk. For the INCT design, restarting the RP at the beginning of each stage may be beneficial, as tests are conducted independently at each stage rather than cumulatively across all previously allocated patients, as is the case in the LDM design.

Randomization procedures differ in their approach to treatment allocation. While some procedures do not aim for a final balance, others aim for a balance at the end of the allocation process or at defined interim steps. It would be helpful to classify and differentiate between the procedures under consideration in terms of balance, also to be able to assess further procedures. It goes without saying that there are other procedures.

Answer: We fully agree. We have revised the Discussion section to include a classification of the randomization procedures evaluated in our study, based on their balance properties and reference

the randomizeR package toexplain how users can evaluate further procedures using our provided code.

Our simulation shows, that c~~C~~omplete randomization, though conceptually simple, leads to a ~~substantial~~ power loss for small sample GSDs~~, as observed in our simulations~~. To mitigate this issue, restricted randomization methods ~~like PBR~~ are commonly employed to achieve balanced treatment groups sizes with respect to final analysis (final balance) as well as stage analysis (stage-wise balance). If no further restriction, like stratification of the RP by stage is implemented, the only procedure which achieves stage-wise balance is PBR by using according block sizes. However, PBR in unblinded or single-blinded trials, which are common in fields such as rare diseases [23], carries a ~~significant~~ high risk of predictability [24]. For example, in a trial with a maximum sample size of $n = 24$ ~~and $K = 3$ stages~~, an investigator could correctly predict at least 1/4 of the allocations with certainty, if he correctly assumes that PBR(4) was used, the most common used RP and block size in our literature review on GSDs, or simply follows the "convergence strategy" for guessing the next treatment allocation [26]. This predictability introduces a ~~substantial~~ risk of allocation bias, which could undermine the trials validity.

RAR, a special form of block randomization with the block size being the total sample size, can keep the final balance, but does not maintain the stage-wise balance. This procedure was shown in our simulations to perform sub-optimally in regards to power for INCT in small sample GSDs, as it does not keep the stage-wise balance. Other RPs, like BSD and CHEN keep a maximum tolerated imbalance, where the imbalance at each time of the allocation process never exceeds a certain threshold. This is particularly useful in small sample clinical trials, as higher deviations from the stage-wise 1 : 1 are prohibited.

The last category of RPs are those that can become arbitrarily imbalanced, which is very problematic in small sample group sequential clinical trials, namely CR and EBC.

It should be noted, that we evaluated some special candidates of RPs, showing the property of stage-wise balance, final balance only, keeping the maximal tolerated imbalance only, as well as having no restrictions, to mirror the picture of these properties. The randomizeR package can generate sequences for other RPs [40], and these can be used together with our code [45] to evaluate further RPs.

Minor comments

"Simulation results demonstrate that deficiencies in in the implementation of randomization can inflate type I error rates." What exactly is meant by "deficiencies"? It would be good to see at least one example of this shortcoming and possible measures to prevent it.

Answer: We added an example at the beginning of the Results section explaining how using predefined boundaries without adjusting for actual allocation imbalance can inflate the type I error.

Results of the simulation study for the z-test

Consider the following scenario: In a trial with a GSD, the critical boundaries are specified in the study protocol based on the (planned) stage-wise sample size and number of stages. These predefined boundaries are then naively applied during the analysis, without accounting for the stage-wise deviations in the observed allocation ratio due to the RP. Fig 3 illustrates this scenario with a violin plot depicting the T1E for a maximum sample size of $n = 24$ and 2 interim analyses for different RPs. This increase in T1E occurs when the intended allocation ratio is not maintained for every stage of the trial.

The description in Table 1 is (mainly) based on the case of two treatments. This should be made explicit or the description should be at a general level.

Answer: We agree with your suggestion. We have updated the title of Table 1 to "Glossary of important terms related to randomization in 2-arm clinical trials comparing two treatments."

Table 1. Glossary of important terms related to randomization in 2-arm clinical trials comparing two treatments.

Term	Description

The term “sequence” should be added to Table 1

Answer: We have added the term "randomization sequence" to Table 1 as recommended.

Randomization sequence	The order in which participants are assigned to treatment groups in a randomized clinical trial. The sequence is typically generated from a randomization procedure to ensure unbiased treatment allocation [8].

“In the case of randomized PBR” should be explained. Is permuted block randomization with varying block sizes meant here?

Answer: Yes, this refers to permuted block randomization with block sizes randomly selected from a predefined set. We have clarified this term in the glossary.

Randomized Permuted Block Randomization	Permuted block randomization with block sizes for each block randomly selected from a predefined set [25].

“This is important because an imbalance in one stage can reverse in a later stage, resulting in 2a consecutive allocations to the same group.” What is meant by “2a”?

Answer: We have revised the sentence for clarity. The updated sentence now reads:

Additionally, when using RPs that allow a maximum tolerated imbalance a, this maximum tolerated imbalance should be smaller than the smallest stage size for LDM and less than half the smallest stage size for INCT. ~~This is important because an imbalance in one stage can reverse in a later stage, resulting in $2a$ consecutive allocations to the same group.~~ This requirement is necessary, because once an imbalance of a is reached in one direction, the allocation rule allows up to $2a$ consecutive allocations to the opposite group.

Page 12, line 333: “Table 6 provides a framework indicating suiTable RPs to pair with GSDs for trials with small, stage-wise sample sizes.” – typo T in suiTable

Answer: Corrected.
